# Current knowledge and breeding strategies for management of aphid-transmitted viruses of pepper (*Capsicum* spp.) in Africa

**DOI:** 10.3389/fpls.2024.1449889

**Published:** 2024-10-25

**Authors:** Herbaud P. F. Zohoungbogbo, Fabrice Vihou, Enoch G. Achigan-Dako, Derek W. Barchenger

**Affiliations:** ^1^ World Vegetable Center, West and Central Africa–Coastal and Humid Regions, Cotonou, Benin; ^2^ Genetics, Biotechnology and Seed Science Unit, Laboratory of Crop Production, Physiology and Plant Breeding, Faculty of Agronomic Sciences, University of Abomey-Calavi, Abomey-Calavi, Benin; ^3^ World Vegetable Center, Tainan, Taiwan

**Keywords:** host resistance, integrated pest management, Potyvirus, Cucumovirus, Polerovirus

## Abstract

Aphid-transmitted viruses cause significant losses in pepper production worldwide, negatively affecting yield and quality. The emergence of new aphid-transmitted viruses or development of variants as well as the occurrence in mixed infections make management a challenge. Here, we overview the current status of the distribution, incidence and phylogeny of aphids and the viruses they transmit in pepper in Africa; outline the available genetic resources, including sources of resistance, resistance genes and molecular markers; and discuss the recent advances in understanding the genetic basis of resistance to the predominant African viruses infecting pepper. *Pepper veinal mottle virus* (PVMV; *Potyvirus*); *Potato virus Y* (PVY; *Potyvirus*), *Chili veinal mottle virus* (ChiVMV; *Potyvirus*), *Cucumber mosaic virus* (CMV; *Cucumovirus*) and *Pepper veins yellow virus* (PeVYV; *Polerovirus*) have been reported to be the most widespread and devastating aphid-transmitted viruses infecting pepper across Africa. Co-infection or mixed infection between aphid-transmitted viruses has been detected and the interrelationship between viruses that co-infect chili peppers is poorly understood. Establishing and evaluating existing and new diversity sets with more genetic diversity is an important component of developing host resistance and implementing integrated management strategies. However, more work needs to be done to characterize the aphid-transmitted viral strains across Africa and understand their phylogeny in order to develop more durable host resistance. In addition, a limited number of QTLs associated with resistance to the aphid-transmitted virus have been reported and QTL data are only available for PVY, ChiVMV and CMV mainly against European and Asian strains, although PVMV is likely the most important aphid-transmitted viral disease in Africa. There is a need to identify germplasm resources with resistance against various aphid-transmitted virus strains, and subsequent pyramiding of the resistance using marker-assisted selection could be an effective strategy. The recent advances in understanding the genetic basis of the resistance to the virus and the new breeding techniques that can be leveraged to accelerate breeding for aphid-transmitted virus in pepper are proposed as strategies to more efficiently develop resistant cultivars. The deployment of multi-genetic resistances in pepper is an effective and desirable method of managing viral-diseases in Africa and limit losses for farmers in a sustainable manner.

## 1 Introduction

Pepper (*Capsicum* spp.) is an important Solanaceae crop cultivated for culinary use as vegetable and spice, and a source of food colorants and secondary metabolites (Mimura et al., 2012). Importantly, being a high-value crop, pepper is a source of income for farmers, especially smallholder farmers in Asia and Africa (Islam et al., 2020; Waweru et al., 2020b). Moreover, Africa contributes 1,008,574 tons (approximately 21% of global production) with a harvested area of 375,989 ha of global dry pepper production, and 3,472,485 tons (approximately 10% of global production) for a harvested area of 331,064 ha of global green pepper production (FAOSTAT, 2022). The genus *Capsicum* includes 43 species, among which five are domesticated and cultivated (Barboza et al., 2022). The domesticated and widely cultivated species are *C. annuum* (L.), *C. frutescens* (L.), *C. chinense* (Jacq.), *C. baccatum* (L.), and *C. pubescens* (Ruiz and Pav.) (da Costa et al., 2006; Ibiza et al., 2012). Globally, *C*. *annuum* is the most widely produced and consumed species; however, in Africa, significant production of *C*. *chinense* and to a lesser extent *C*. *frutescens* also occurs (Zohoungbogbo et al., 2024).

In Africa, chili production is generally limited to small areas that range from 0.5 to 1.2 ha (Segnou et al., 2012; Dagnoko et al., 2013; Zohoungbogbo et al., 2024), and the crop can be an important source of income for smallholder and family farmers in rural areas. However, pepper production is frequently threatened by many biotic factors, such as diseases, weeds, and pests. Among the biotic stresses causing losses for producers in Africa, viral diseases are reported to be the most significant constraint to pepper production (Zohoungbogbo et al., 2022). The increasing outbreaks of viral species infecting pepper has become a major problem for growers across Africa (Arogundade et al., 2020; Waweru et al., 2021; Zohoungbogbo et al., 2022). Most of the viruses that infect pepper are transmitted by arthropod vectors namely aphids, whiteflies or thrips, and as such farmers mainly rely heavily on insecticides to manage them (Schreinemachers et al., 2015; Zohoungbogbo et al., 2024). In Africa, numerous viruses are reported to cause symptoms in pepper, including *Alfalfa mosaic virus* (AMV*; Alfamovirus*), *Blackeye cowpea mosaic virus* (BICMV; *Potyvirus*), *Chili Veinal Mottle Virus* (ChiVMV; *Potyvirus*), *Cowpea aphid borne mosaic virus (*CABMV*; Potyvirus), Cucumber mosaic virus* (CMV; *Cucumovirus*), *Pepper veinal mottle virus* (PVMV; *Potyvirus*), *Pepper vein yellows virus* (PeVYV; *Polerovirus*), *Pepper mild mottle virus (*PMMoV*; Tobamovirus*), *Potato virus* X (PVX; *Potyvirus*), *Potato virus Y* (PVY; *Potyvirus*), *Tomato yellow leaf curl virus* (TYLCV*; Begomovirus*), *Tobacco etch virus (*TEV*; Potyvirus)*, *Tobacco mosaic virus* (TMV; *Tobamovirus*), *Tomato mosaic virus* (ToMV; *Tobamovirus*), *Tobacco mild green mosaic virus* (TMGMV; *Tobamovirus*), and *Tomato spotted wilt virus* (TSWV; *Tospovirus*) (Karavina et al., 2016; Waweru et al., 2021; Zohoungbogbo et al., 2022).

Aphids are an important vector of pepper viruses in Africa and cause significant economic losses (Waweru et al., 2021). The cotton aphid (*Aphis gossypii* Glover), green peach aphid (*Myzus persicae* Sulz) and potato aphid (*Macrosiphum euphorbiae*) are the most efficient vectors of plant viruses in pepper (Weintraub, 2007); however, the population of aphids in Africa has not been widely studied. Farmers often use insecticides to control aphids to reduce their numbers before they damage crops (Hooks and Fereres, 2006). However, this approach was found to be ineffective in reducing the spread of nonpersistent viruses because the viruses are transmitted during probing, often before farmers observe the presence of aphids in their field (Fereres, 2000). Furthermore, aphids are a serious pest during seedling nursery production under protected cultivation, prior to transplanting, these viruses can be transmitted before the plants entering the field (Latifah et al., 2021). Once in the field, farmers primarily depend on insecticide applications to manage aphid populations and minimize crop damage (Hooks and Fereres, 2006). However, this approach has proven ineffective in reducing the spread of aphid-transmitted viruses, as these viruses are transmitted before the insecticides have a chance to kill the aphids (Fereres, 2000). In addition, many aphid species have developed intolerance to the various pesticides commonly deployed by producers (Li and Han, 2004). The use of virus-resistant varieties in combination with other cultural practices appears to be among the most promising strategies to control aphid-transmitted viruses in pepper. Resistant varieties are highly preferred because they can reduce the virus incidence in the fields and thus the virus inoculum in the farming system and they are also compatible with other control methods (Frantz et al., 2004; Waweru et al., 2021). Breeding for host resistance to multiple and diverse viruses is required for sustained pepper production (Wiesner-Hanks and Nelson, 2016) and can be achieved by the pyramiding of disease resistance genes through the use of marker-assisted selection (MAS) and backcrossing or the development of specialized breeding populations such as recombinant inbred lines (RIL), introgression line (IL) or multiparent advanced generation inter-cross (MAGIC) populations.

Despite the fact that viral diseases are one of the major constraints, causing significant yield losses in peppers in Africa (Olawale et al., 2015; Zohoungbogbo et al., 2024), there is a lack of recent comprehensive reviews focusing on the most significant viruses impacting pepper production in the different regions of Africa. The objectives of this review are to (1) present an overview of the current status of the distribution, incidence and phylogeny of aphids and the viruses they transmit in pepper in Africa; (2) outline the available genetic resources, including sources of resistance, resistance genes and molecular markers linked to resistance genes; and (3) discuss the recent advances in understanding the genetic basis of resistance to the predominant African viruses infecting pepper and the breeding techniques that can be leveraged to accelerate breeding for resistance to aphid-transmitted viruses of pepper.

## 2 Distribution, symptoms and incidence of aphid-transmitted viruses in pepper in Africa

**Figure 1:**
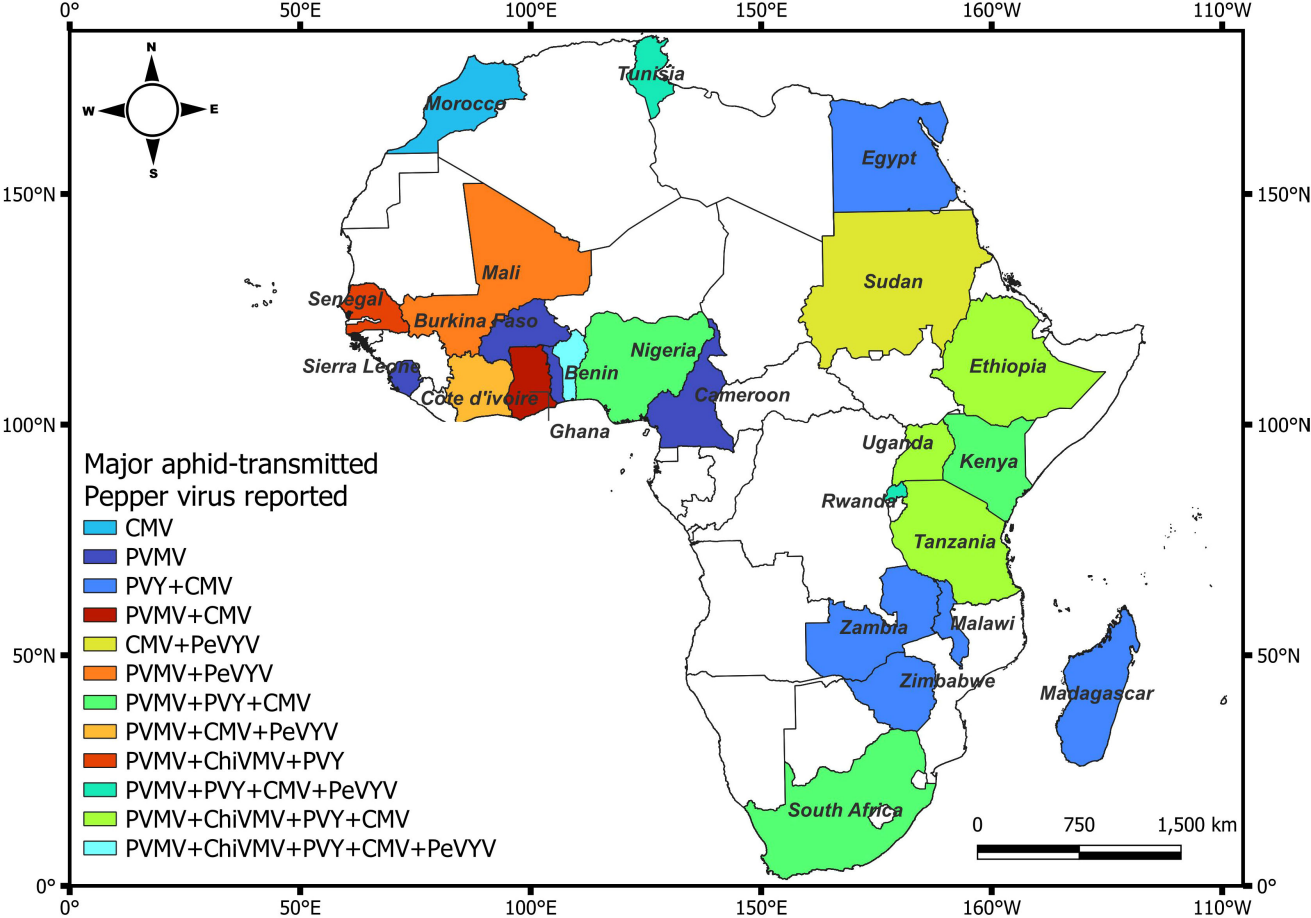
Distribution of major aphid-transmitted viruses of pepper in single as well as in mixed infection across Africa. Countries in white do not have reports of aphid-transmitted viruses. Citations of the reports of the aphid-transmitted viruses in pepper are listed in the **Supplementary Table 1**.

*Pepper veinal mottle virus* (PVMV), *Cucumber mosaic virus* (CMV), *Potato virus Y* (PVY), *Chili Veinal Mottle Virus* (ChiVMV) and *Pepper vein yellows virus* (PeVYV) are the most predominant aphid-transmitted viruses affecting pepper in Africa (Waweru et al., 2021; Zohoungbogbo et al., 2022). The prevalence of these viruses may be attributed to their broad host range and the fact that they can be transmitted by several species of aphids (Pernezny et al., 2003). Viruses belonging to the genus *Potyvirus* (PVMV, ChiVMV and PVY), *Cucumovirus* (CMV) and *Polerovirus* (PeVYV) have been reported to cause symptoms in pepper in in West, East and Southern Africa (**Figure 1**). To date, PVMV was reported in at least 17 countries in Africa (**Figure 1**; **Supplementary Table 1**). *Cucumber mosaic virus* was reported in at least 19 countries in Africa, and causing significant losses in pepper worldwide. Similarly, PVY causes significant losses across Europe and has been reported in at least 16 countries in Africa (**Figure 1**; **Supplementary Table 1**). Although a major threat to pepper production in Asia, ChiVMV has also been reported in at least five African countries (**Figure 1**; **Supplementary Table 1**). An emerging threat to pepper in Asia and in at least six countries in Africa is PeVYV, which has only recently been identified (Knierim et al., 2013). Historically, PVY, and more recently CMV and PVMV are the most important viruses reported among the aphid transmitted viruses in Africa (Bolou Bi et al., 2018; Waweru et al., 2019; Adediji et al., 2021).

### 2.1 Potyvirus infecting pepper in Africa

Members of *Potyvirus* are single stranded RNA viruses that are polyadenylated at the 3’ end (A(n)) and have a covalently linked VPg (viral protein genome-linked) protein at the 5’ end (Tennant et al., 2018). The VPg protein helps the virus to replicate and to infect plant cells (Tennant et al., 2018). The *Potyvirus* genome has one open reading frame (ORF), which is translated into a large polyprotein that is then cleaved into individual proteins by three virus-encoded proteases: P1(proteinase), HC-Pro (helper component-proteinase), and NIa (nuclear inclusion protein a). One of the proteins produced from the polyprotein is P3N-PIPO, which is a fusion protein of the N-terminal end of P3 (component of viral replication complexes) and a translation frameshift product derived from a hidden pipo cistron (Tennant et al., 2018). Another protein produced from the polyprotein is PISPO (Pretty Interesting Sweet Potato Potyvirus ORF), which is a 230-codon protein that is specific to some sweet potato-infecting potyviruses (Tennant et al., 2018).

**Figure 2:**
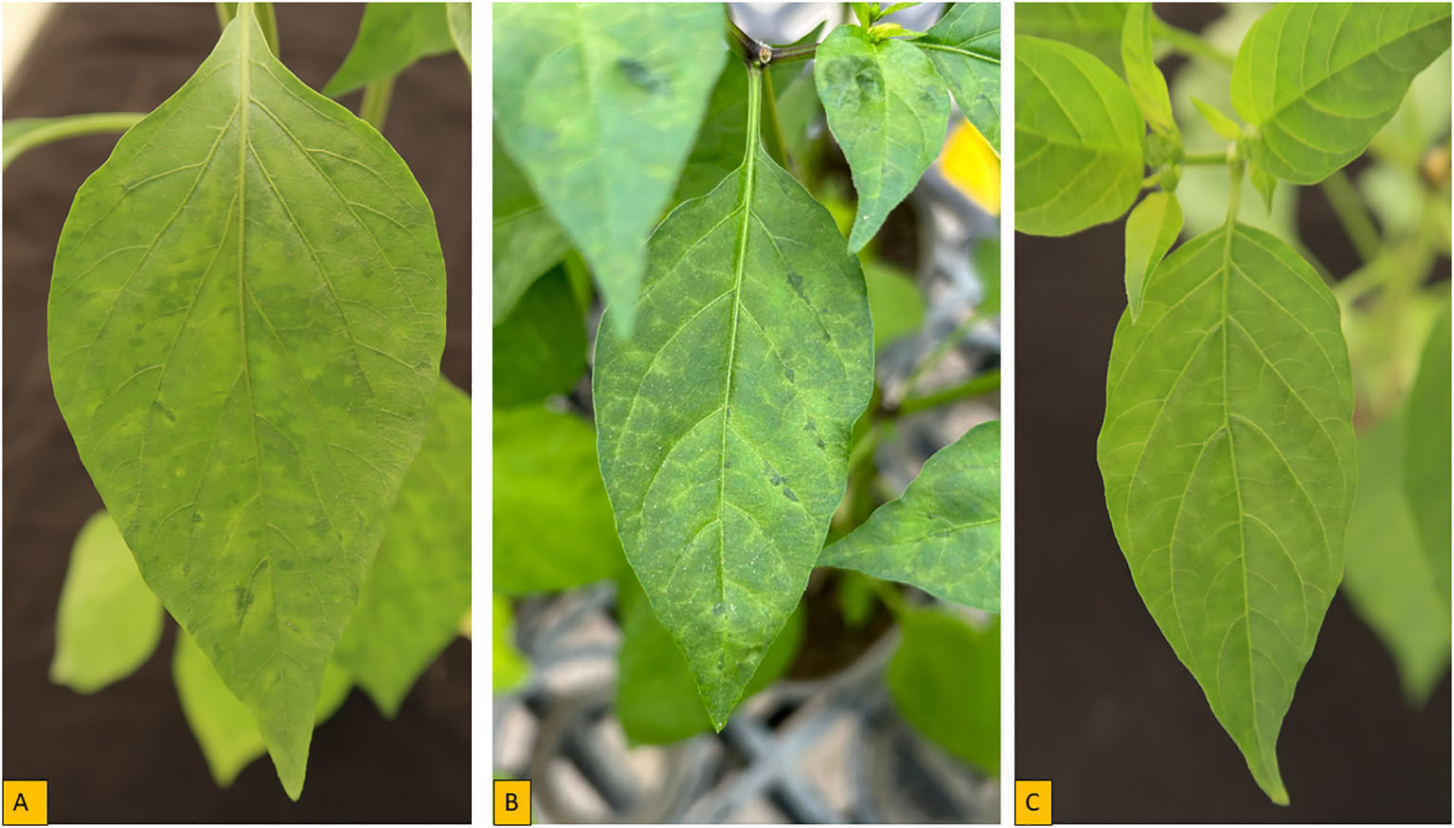
Symptoms of pepper plant inoculated with PVMV- S-0002 isolate from eggplant in controlled environment. **(A)** mosaic **(B)** vein banding **(C)** Mottle.

The genus *Potyvirus* contains over 180 distinct viruses (Barka and Lee, 2020) and in Africa, potyviruses are among the most threatening viruses to pepper production. *Pepper veinal mottle virus* was first identified in pepper in 1971 by Brunt and Kenten (1971) in Ghana in West Africa and was prevalent in both *C. annuum* and *C. frutescens* cultivars collected in the region. Now, PVMV is widely distributed across West African countries and represents the major virus in many countries in this region of Africa (Huguenot et al., 1996; Afouda et al., 2013; Kenyon et al., 2014; Bolou Bi et al., 2018; Adediji et al., 2021). Originally, PVMV was considered a member of the PVY group (Brunt and Kenten, 1971; De Wijs, 1973). It has been reported that PVMV causes significant economic losses as a result of reduced yield of 54.5-64.3%, and very high incidence of up to 100% have been observed in pepper fields in Nigeria (Alegbejo and Abo, 2002; Fajinmi et al., 2012). However, ecological characteristics, climate and vegetation (as hosts) in the different ecological conditions appeared to play a major role in determining the incidence and severity of PVMV infection on pepper in the fields (Bolou Bi et al., 2018). The severity of PVMV also depends on the plant cultivar, crop management and the stage of growth of the crop at which infection occurred (Soh and Yap, 1977). Several symptoms are associated with PVMV on infected pepper (**Figure 2**), including chlorosis, necrosis, mottling, chlorosis, deformed leaves with leaf puckering or curling (Kenyon et al., 2014; Adediji et al., 2021).

*Potato Virus Y* has previously been reported in many African countries (Thompson et al., 1987; Foster and Mills, 1990; Budnik et al., 1996), resulting in a yield reduction of 20-70% in pepper production (Waweru et al., 2019). Recently, the incidence of PVY in Africa seems to be decreasing, for example, Bolou Bi et al. (2018) and Zohoungbogbo et al. (2024), did not detect the presence of this virus in their country-wide diagnosis in Côte d’Ivoire and Benin, respectively. The most common symptoms induced by PVY in pepper include stunting or dwarfing of the plant, systemic vein clearing and banding, leaf mosaic and small deformed fruits with a mosaic pattern making them unmarketable (Dogimont et al., 1996; Arogundade et al., 2020). In some extreme cases and depending on the pepper cultivar, the strain of PVY, environmental conditions, and the time of infection, necrotic spots, mosaic patterns and distortions may develop on fruit stem and apical bud necrosis can lead to plant death (Moury and Verdin, 2012). Yield losses greatly depend on the growth stage of the plant at time of infection and can reach up to 100% (Avilla et al., 1997). In some West African countries, such as Nigeria (especially in southwest Nigeria), incidence of PVY a decade ago was the highest (79%) compared to TEV (67%), CMV (61%), and PVMV (58%) and lowest for ToMV (23%) (Olawale et al., 2015). However, few reports of PVY being a threat to African pepper production have been published more recently (**Figure 1**; **Supplementary Table 1**).

**Figure 3:**
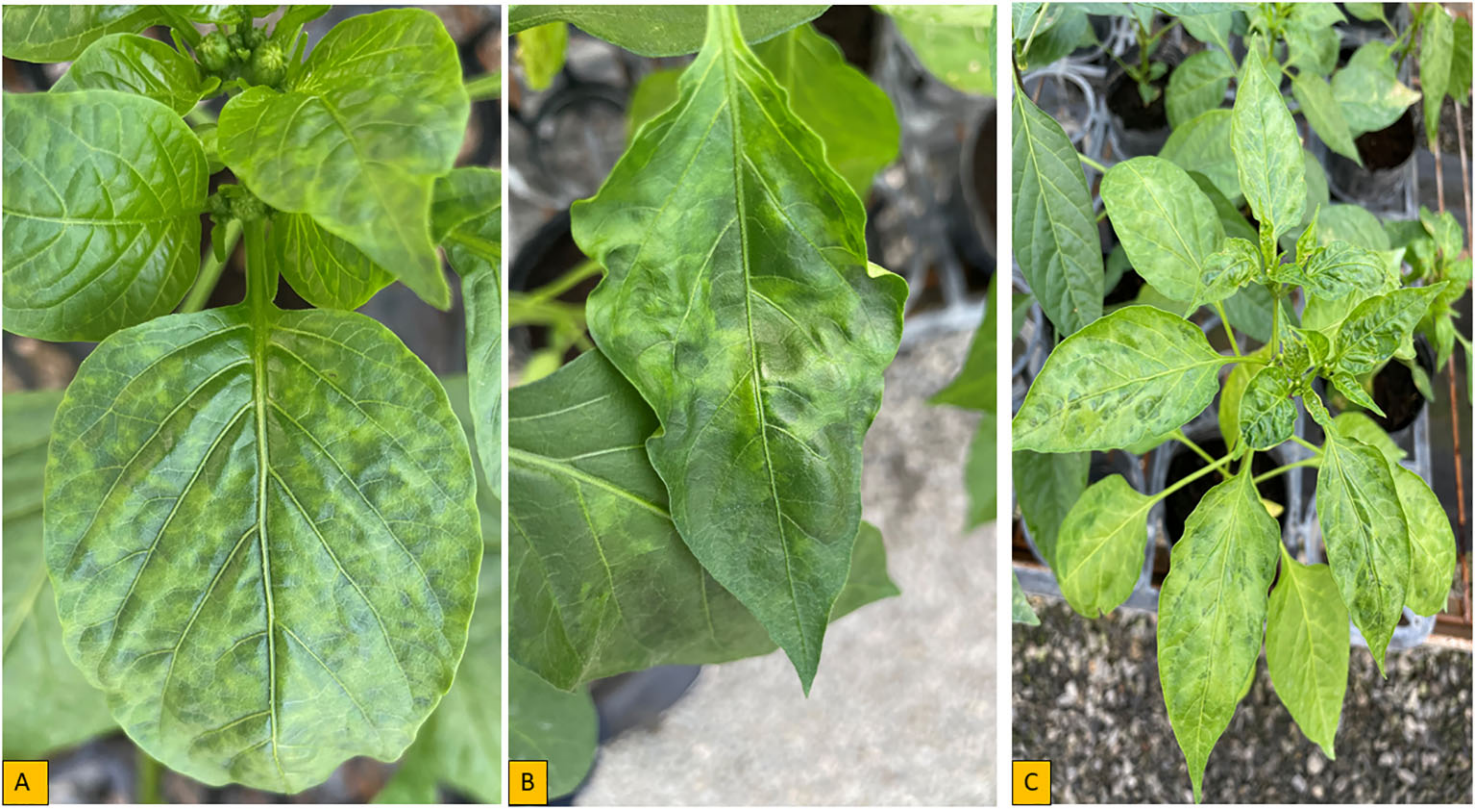
Symptoms of pepper plant inoculated with ChiVMV isolate in controlled environment **(A)** Interveinal yellowing, **(B, C)** Severe mosaic with leaf deformation, wavy and blistering.

*Chili Veinal Mottle Virus* was first reported in Malaysia and has since spread and resulted in decreased pepper productivity in Asia and Africa (Green and Kim, 1994; Lee et al., 2013). Disease caused by ChiVMV has been reported to result in a 30% reduction in pepper yields in 16 Asian countries on average (Lee et al., 2017). *Chili veinal mottle virus* has been reported in various countries in West and East Africa. The virus has been found in five African countries including Benin, Senegal, Ethiopia, Uganda and Tanzania (Dafalla, 2001; Moury et al., 2005; Zohoungbogbo et al., 2022). Typical symptoms caused by ChiVMV include leaf mottling, dark green vein-banding, vein-clearing, and leaf chlorosis (**Figure 3**) (Tsai et al., 2008; Arogundade et al., 2020). Among 16 host plants tested by Reddy et al. (2022), seven species known as tobacco (*Nicotiana tabacum* cv. Samsun), tobacco (*N. glutinosa*), Western Nimba Toad (*N. occidentalis*), thorn apple (*Datura metel*), *Physalis floridana*, African nightshade (S*olanum nigrum*), and pepper) were infected with the ChiVMV disease and the symptom could be seen in 20-25 days after inoculation.

### 2.2 Cucumovirus infecting pepper in Africa

*Cucumber mosaic virus*, the type member of the genus *Cucumovirus*, has a very wide host range and is one of the most prevalent viruses of pepper worldwide (Ali and Kobayashi, 2010). The virus was first described in cucumbers (*Cucumis sativus* L.) in Michigan, United States in 1916 (Doolittle, 1920). The genome of CMV is made up of three single-stranded positive-sense RNA (ssRNA) molecules, all of which are positive polarity, each separately encapsidated in 29-nm diameter icosahedral virions (Kenyon et al., 2014). Each molecule has a 5’ cap at one end and a tRNA-like structure at the other end (Tennant et al., 2018). Genomic RNA1 contains the gene for a replicase protein (P1a) that has methyltransferase (Mtr) and helicase domains (Salánki et al., 2018; Tennant et al., 2018), which are essential for initiating replication. Genomic RNA2 contains the gene for a second replicase protein (P2a) that has a polymerase domain (RdRp), which is essential for copying the viral RNA. Genomic RNA3 contains the gene for a fourth nonstructural protein, the movement protein (P3a), and the structural capsid protein (P3b) (Salánki et al., 2018; Tennant et al., 2018). The P3a gene is involved in spreading the virus from cell to cell, and that makes up the capsid, or shell, of the virus particle (Salánki et al., 2018; Tennant et al., 2018). In addition to these three genomic RNAs, CMV also produces subgenomic RNAs (sgRNAs), which are shorter than genomic RNAs and contain only a subset of the genes (Salánki et al., 2018; Tennant et al., 2018).

**Figure 4:**
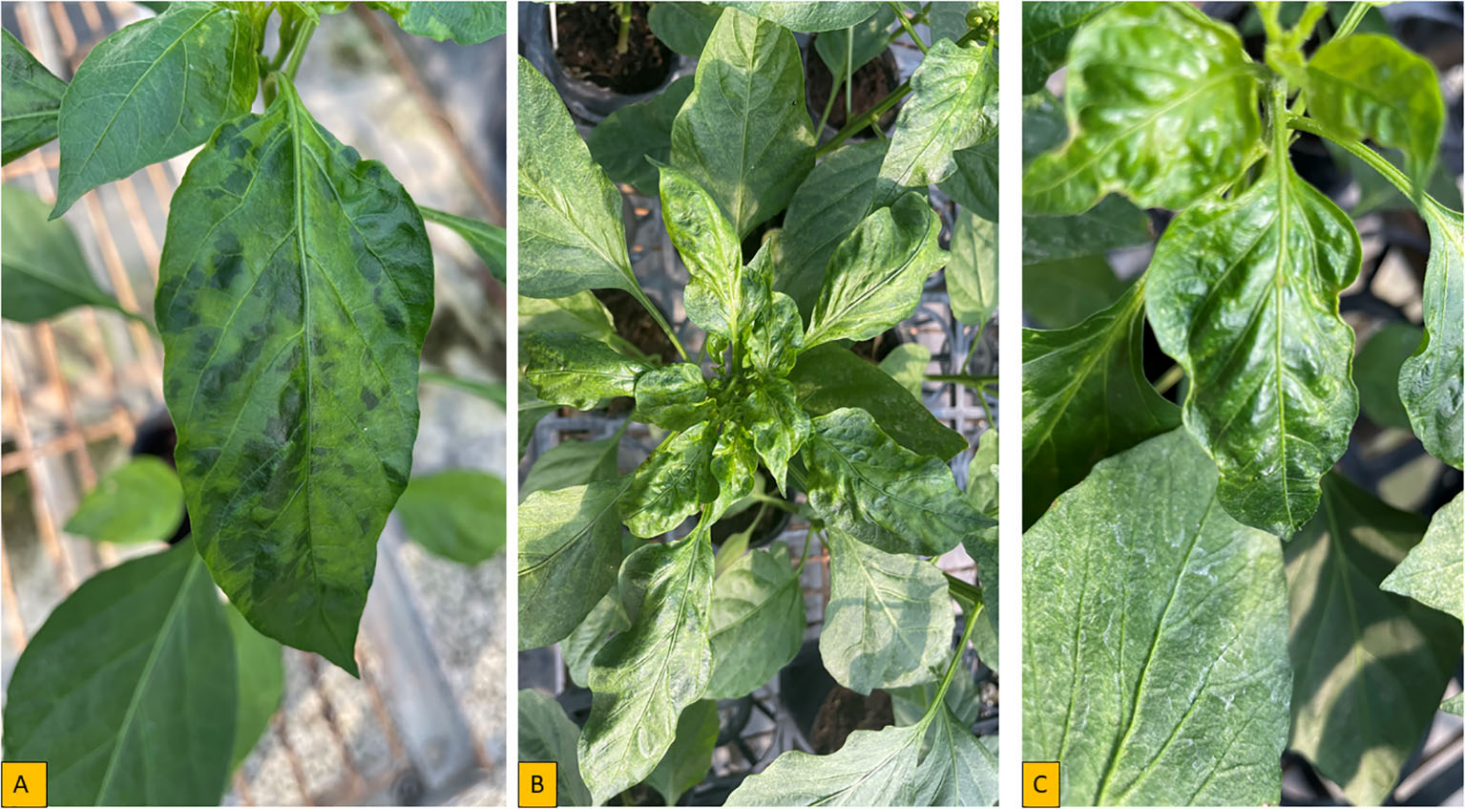
Symptoms of pepper plant inoculated with CMV isolate in controlled environment **(A)** Severe mosaic **(B)** Severe mosaic with leaf deformation **(C)** Wavy.

*Cucumber Mosaic Virus* was reported in at least 19 countries in Africa and represents a very important virus in pepper on the continent (**Figure 1**; **Supplementary Table 1**). Several strains of pepper-infecting CMV exist, which differ in their symptom expression (Green and Kim, 1994). The age of a plant at the time of infection strongly influences symptoms manifestation (Murphy and Bowen, 2006). Symptoms of CMV vary, but the most prominent symptoms include mild mosaic and dull-colored leaves, mottling, shoe string, fern leaf, vein banding, vein clearing, lead deformation, stunted growth and reduced fruit size (**Figure 4**). Aphids are the most important means of CMV transmission (Edwardson and Christie, 1986); however, transmission can also occur through seed sourced from infected mother plants, parasitic weeds such as Egyptian broomrape (*Orobanche aegyptiaca*) (Aly, 2013) and also mechanically (Ali and Kobayashi, 2010; Jacquemond, 2012).

### 2.3 Polerovirus infecting pepper in Africa

The genus *Polerovirus* contains twenty-six virus species that infect a wide variety of plants from cereals to cucurbits and peppers (LaTourrette et al., 2021). In Africa, the first reports of PeVYV were in 2013 in Mali and Tunisia (Knierim et al., 2013). The history of PeVYV began with bell pepper plants, grown in a greenhouse, with vein yellowing and leafroll symptoms at Kitanakagusuku, Okinawa Prefecture, Japan in 1981 (Yonaha et al., 1995). Based on symptomatology, the causal agent was clearly different from TMV, CMV, *Broad bean wilt virus* (BBVW; *Fabavirus*), *Chrysanthemum mild mottle virus* (CMMV; *Cucumovirus*), PVY and TSWV, which had been previously isolated from pepper plants in Japan (Yonaha et al., 1995). The causal agent was classified as a new member of the *Luteovirus* group and named PeVYV. In 2011, the complete genomic sequence of PeVYV was studied, which led to PeVYV being classified as a *Polerovirus* (Murakami et al., 2011). *Pepper vein yellows virus* has been reported now in six African countries including Côte d’Ivoire, Benin, Mali, Tunisia, Rwanda and Sudan (Buzkan et al., 2013; Knierim et al., 2013; Alfaro-Fernández et al., 2014; Bolou et al., 2015; Waweru et al., 2021) (**Figure 1**) with an infection up to 100% in some pepper fields (Tomassoli et al., 2016).

*Pepper vein yellows virus* is spread in circulative and non-propagative manner by *A. gossypii* and *M. persicae* (Murakami and Kawano, 2017). Hosts of PeVYV include pepper, African nightshade, tobacco among other crop plants (Knierim et al., 2013; Alabi et al., 2015; Wang et al., 2017). Symptoms of PeVYV on pepper include leaf curling, deformation, reduced leaf size, puckering, interveinal yellowing, vein clearing, and yellow patches on leaves, shortening of stem internodes, upward curling of the leaf blade and small, discolored fruit (Dombrovsky et al., 2010; Panno et al., 2016). Frequent mixed infection of PeVYV with other viruses is common, including other important aphid-transmitted viruses such as CMV and PVMV (Knierim et al., 2013; Zohoungbogbo et al., 2022) and also a synergistic effect of mixed infection between members of *Begomovirus* and *Polerovirus* as reported (Koeda et al., 2020). Koeda et al. (2020) reported that studies should investigate the pathogenicity of PeVYV-9 and host range and the effect of mixed infections with begomoviruses.

Poleroviruses have a 5.2 to 6.3 kb positive-sense RNA genome from which a subgenomic mRNA (sgRNA1) is generated in infected cells (Kelly et al., 1994). *Pepper vein yellows virus* genomes consist of single linear, positive-sense, single-stranded RNA containing 6,244 nucleotides (nt), including six open reading frames (ORFs; ORF0 to ORF5) (Liu et al., 2016; Wang et al., 2021). *Poleroviruses* produce two subgenomic RNA molecules during replication, which are short (5.2 to 6.3 kb) RNA molecules that contain only a subset of the genes from the full-length viral genome (Smirnova et al., 2015). The subgenomic RNA molecules are translated into different proteins using several different mechanisms (LaTourrette et al., 2021).

## 3 Co-infection of aphid-transmitted viruses and their interaction in pepper

Co-infection or mixed infection between pepper viruses has been detected in almost all countries in Africa where aphids transmitted viruses have been reported (Fatogoma et al., 2014; Olawale et al., 2015; Waweru et al., 2021). Mixed infection causes synergistic or antagonistic interactions of the viruses in the plant (Syller, 2012). There has been a wide body of research conducted to understand the effect of the co-infection interaction and the implication in the disease severity (Megahed et al., 2019; Singhal et al., 2021; Vinodhini et al., 2021; Ontiveros et al., 2022). For instance, when tobacco plants are infected with both PVY and PVX, the symptoms were more severe and the PVX titer in the plant increased up to 10 times compared to when the plants were infected with only a single virus (Rochow and Ross, 1955). Wintermantel et al. (2008) reported that when co-infection of TMV and CMV in benthi (*N. benthamiana*) plants occurred, the amount of TMV increased and the amount of CMV decreased, compared to when the plants were infected with either virus alone. In most cases of synergistic interaction between a *Potyvirus* and a heterologous virus, the *Potyvirus* titer remains the same or decreases slightly, while the amount of the non-*Potyvirus* titer increases (Pruss et al., 1997). Damiri (2014) found that when pepper plants were infected with a mixture of three viruses (CMV, PVY, and TMV), the height of the plants increased, but the plant biomass and yield significantly decreased. The authors reported that double mixed infection with CMV + TMV or CMV + PVY caused the greatest reduction in yield (52% and 49%, respectively). The interrelationship between viruses that co-infect peppers is still not fully understood. Therefore, it is important that research be conducted to understand the interrelationship between different viruses in co- and mixed-infection. Understanding the effects of co- and mixed-infection of viruses in pepper would allow for better decision making for farmers in disease management, and support breeding for host resistance research.

## 4 The vectors in Africa

Approximately 15 aphid species have been reported to transmit viruses in pepper in Africa (**Table 1**). The majority of the aphid species were reported in West Africa, while the number of species of aphids reported in East and Southern Africa is limited (**Table 1**). Given that several aphid-transmitted viruses have been reported in Eastern and Southern Africa (**Table 1**), it is important to study the diversity of the vector to support better management strategies. The cotton aphid and the green peach aphid are known for transmitting a majority of aphid-transmitted viruses in pepper. The green peach aphid is the most efficient vector of PVY among more than 50 aphid species identified to transmit the virus in a non-persistent manner (Kanavaki et al., 2006). More than 80 species of aphids vector can transmit CMV in a non-persistent manner, but the melon and cotton aphid and the green peach aphid are the most efficient (Li et al., 2020). For ChiVMV, the melon and cotton aphid, the green peach aphid, the black legume aphid (*A. craccivora* C.L.Koch), the green citrus aphid (*A. spiraeeola* Patch), the brown citrus aphid *(Toxoptera citricidus* Kirkaldy), the corn aphid *(Rhopalosiphum maidis* Fitch), and the rusty plum aphid (*Hysteroneura setariae* Thomas) retain ChiVMV for not more than one hour after the virus acquisition (Arogundade et al., 2020). The green peach aphid and the melon cotton aphid vector PeVYV, in a persistent manner (Yonaha et al., 1995; Murakami et al., 2011).

**Table 1:** Aphid species vectoring viruses reported on pepper in Africa.

Aphid common name	Species	Virus vectored	Countries	Reference
Cotton/melon aphid	*Aphis. gossypii*	PVMV, CMV, ChiVMV, PVY, PeVYV.	Benin, Nigeria, Côte d’Ivoire, Rwanda	De Wijs (1973); Fajinmi et al. (2011); Sæthre et al. (2011); Salaudeen et al. (2018); Waweru et al. (2021); Yonaha et al. (1995)
Greenfly	*Myzus persicae*	PVY, CMV, ChiVMV	Ghana, Nigeria, Senegal, Ethiopia, Zimbabwe	Collingwood et al. (1981); Fajinmi et al. (2011); Karavina et al. (2016); Murage et al. (2016)
Black legume aphid	*Aphis craccivora*	ChiVMV	Benin, Nigeria	Fajinmi et al. (2011); Sæthre et al. (2011); Salaudeen et al. (2018)
Black bean aphid	*Aphis fabae*	CMV, PVY	Nigeria, Ethiopia	Fajinmi et al. (2011); Fekadu et al. (2009)
Corn leaf aphid	*Rhopalosiphum maidis*	ChiVMV	Nigeria	Fajinmi et al. (2011)
Green citrus aphid	*Aphis spiraecola*	ChiVMV	Nigeria, Côte d’Ivoire	Fajinmi et al. (2011); De Wijs (1973)
Brown citrus aphid	*Toxoptera citricidus*	ChiVMV	Côte d’Ivoire	De Wijs (1973)
Potato-tomato aphid	*Macrosiphum euphorbiae*	CMV and PVY	Rwanda, Ethiopia	Simon et al. (2009); Waweru et al. (2021)
Lettuce aphid	*Hyperomyzus latucae*	EPMV	Ethiopia	Simon et al. (2009)
Mustard aphid	*Lipaphis erysimi*	EPMV	Ethiopia	Simon et al. (2009)
Bird cherry-oat aphid	*Rhopalosiphum padi*	PVY	Ethiopia	Simon et al. (2009)
Pea aphid	*Acyrthosiphon pisum*	EPMV	Ethiopia	Simon et al. (2009)

PVMV, Pepper veinal mottle virus (Potyvirus); CMV, Cucumber mosaic virus (Cucumovirus); ChiVMV, Chili veinal mottle virus (Potyvirus); PVY, Potato virus Y (Potyvirus); PeVYV, Pepper vein yellows virus (Polerovirus); EPMV, Ethiopian pepper mottle virus (Potyvirus).

## 5 Screening techniques and symptom scoring for aphid-transmitted viruses

### 5.1 Screening techniques

Field screening for resistance to pathogens is generally ineffective, as many plants avoid infection, even under extreme inoculation pressure (Vidavsky et al., 1998). However, field-based screening methods can be useful to preliminarily identify candidates for host resistance, which should be later validated under controlled conditions. Natural screening for resistance is difficult for aphid-transmitted viruses where mixed infection is common (Jones and Naidu, 2019). The considered “hot spots”, which are locations with high disease pressure, with plants/fields infected by aphid-transmitted virus in mixed infection makes the field screening complex. It is therefore important to screen for resistance to aphid-transmitted viruses in pepper in a controlled environment against single isolates or strains of the target virus and to control contamination from aphids and other vectors (Piron et al., 2010). Characterization of the isolates of a particular virus in the target environment using molecular diagnostics tools is essential to facilitate artificial inoculation experiments for host resistance in pepper. Furthermore, understanding viral populations and phylogeny can contribute to understanding and predicting the emergence of resistance breaking strains (Acosta-Leal et al., 2011). It is also important to know the source of the isolates, place of collection, the host of the isolates in order to know the most important hosts present in the region/area and to anticipate a target breeding plan for resistance to the virus. The use of insect-proof facilities and the systematic control of arthropod pests during the disease screening is essential to eliminate confounding factors such as co- or mixed-infection and symptoms of insect feeding, which can make it difficult to score for resistance (Murphy and Bowen, 2006). For screening for host resistance in controlled conditions, plant age should be considered, as the effect of plant development and growth stage can contribute to host resistance (Lapidot, 2007). For most aphid-transmitted viruses in pepper, seedlings should be mechanically inoculated with virus at the two to three true leaves stage of development (Muhyi, 1990). Mechanical inoculation can be accomplished by mixing the inoculum with carborundum powder (silicon carbide), which is applied to the plant with a cotton pad to ensure the virus penetrates the leaf tissue (Shrestha et al., 2014). The procedure for virus screening generally requires a second inoculation, at seven days after the first inoculation to reduce the chance of escapes. Phenotypic data of host reaction can be recorded in terms of symptom manifestation following mechanical inoculation on plants of each cultivar/line, usually one week after the second inoculation and a second scoring can be done after two weeks after the second inoculation (Yeam et al., 2005). The host reaction can also be recorded according to the disease rating scale developed for each of the viruses.

### 5.2 Detection and diagnosis of aphid-transmitted viruses in pepper

Identification of viral diseases by visual observation of the common symptoms can be challenging, because plants can display the same symptoms, typical of viral infection, as in response to unfavorable environmental conditions, nutritional deficiencies and infestation by arthropod pests (Van der Want and Dijkstra, 2006). Thus, several serological-based methods have been used for the diagnosis of the viruses in pepper. To detect aphid-transmitted viruses (PVY, PVMV, PMMoV, CMV, ChiVMV, PeVYV, and TMV) in pepper, two molecular detection methods, enzyme-linked immunosorbent assay (ELISA) and reverse transcription polymerase chain reaction (RT-PCR), have been deployed. The ELISA method is a serological test that uses antibodies to detect the presence of an antigen and can be used to detect both direct (Gorsane et al., 1999; Afouda et al., 2017; Ayo-John and Odedara, 2017; Waweru et al., 2021) and indirect forms of the virus (Phatsaman et al., 2021). For RT-PCR, primers amplifying regions within the RNA-dependent RNA polymerase-encoding sequence of the virus are used for diagnosis (Waweru et al., 2020a). In addition, RT-qPCR can be used to detect titer-load within a plant, which can support the determination resistance level in the host (Nandudu et al., 2024). For example, a plant infected with a particular virus, but with very low amounts of viral titer could be considered as having higher levels of resistance compared to an individual with higher viral titer (Nandudu et al., 2024). This is especially common when symptoms are cryptic, resistance is rare, and there is no host immunity (Shirima et al., 2017). Compared to ELISA, RT-PCR is a more sensitive method and can be used to detect even low levels of viral titer; however, ELISA is less expensive and faster. Monoplex RT-PCR is performed by using cDNA of mixed samples as the template and primers specific for each virus (Bougatef et al., 2005; Gorsane et al., 2005; Banerjee et al., 2014; Thakur et al., 2014; Heo et al., 2021; Gong et al., 2023). Multiplex RT-PCR makes it possible to detect multiple viruses in a single reaction. However, multiplexing involves optimizing and varying the primers concentration and cycling conditions (Nemes and Salánki, 2020; Gong et al., 2023). The choice of method depends on the specific virus being detected and the availability of resources.

## 6 Genetic resources for improvement of aphid -transmitted viruses resistance in pepper

Cultivated and wild relatives of pepper are conserved globally across numerous genebanks around the world, with the largest collection being housed at the World Vegetable Center, Tainan City in Taiwan; however, the most diverse collection, in terms of number of different species, is housed at New Mexico State University (Barchenger and Khoury, 2022). Despite extensive efforts to collect and conserve peppers, limited research has been conducted to systematically evaluate the pepper germplasm for resistance to aphid-transmitted viruses. Some of the seminal research in screening and characterizing host resistance was done by L’institut national de recherche pour l’agriculture, l’alimentation et l’environnement (INRAE) in France (Caranta et al., 1996). Researchers at INRAE worked on the development and study of host resistance to aphid-transmitted viruses such as CMV, PVMV and ChiVMV using techniques like double haploid (DH) or bi parental populations for QTL mapping (Caranta and Palloix, 1996; Caranta et al., 1999, 2002). Resistance to PVMV was identified by Poulos et al. (1973) in two chillies from India, ‘Perennial HDV’ and ‘Pusa Sadabahar PSP-11’ (**Table 2**). Double haploid (DH) lines derived from a cross between pepper varieties Perennial and Yolo Wonder were developed by INRAE (Caranta et al., 1996) and DH801 and 15 breeding lines homozygous for both the *pvr2^2^* and *pvr6* alleles tested negative for PVMV and ChiVMV despite a high prevalence of the PVMV in the surrounding plants in the field trials in Senegal (Moury et al., 2005). Moury et al. (2005) reported that only one isolate of PVMV could infect pepper genotypes carrying the two recessive genes *pvr6* and *pvr1*; however, these genotypes were not infected by PVMV in field trials in Senegal. With the evolution of the PVMV strains and the high infestation reported recently, there is a need to evaluate genotypes with these two recessive genes in Africa.

**Table 2:** Reported pepper sources of resistance to the aphid-transmitted viruses predominant in Africa.

Source	Host species	Virus	Resistance reaction	Isolates tested	Resistance genes/QTLs	Reference
Perennial	*Capsicum annuum*	CMV	Resistant/tolerant	CMV-V26, CMV-V28, CMV-V27, CMV-NY, CMV-Fny and CMV-CA	–	Grube et al. (2000b); Caranta et al. (1996)
		PVMV	Tolerant	Potyvirus (E), Y90/34	*pvr1+ pvr6*	Caranta and Palloix (1996)
		PVY	Completely resistant	Pathotype (0)	*pvr1*	Caranta and Palloix (1996)
			Partially resistant	Pathotype (1,2)	*pvr1*	Caranta et al. (1996)
		ChiVMV	Completely resistant	Isolates from Beijing, Thailand, and Taiwan	*pvr1+ pvr6*	Caranta et al. (1996)
DH801	*C. annuum*	PVMV	Completely resistant	CAC2, CAC3, CAC4, CAC94, F-Bot, S23, S31 Y90/34, potyvirus E	*pvr1* and *pvr6*	Moury et al. (2005)
Florida VR2	*C.annuum*	PVY	Completely resistant	Pathotype (0) and (1)	–	Caranta et al. (1996)
Vania	*C. annuum*	CMV	Completely resistant	CMV-MES and CMV-N	–	Caranta et al. (1996)
PI 439381-1-3	*C. baccatum*	CMV	Resistant	CMV-Y.	–	Suzuki et al. (2003)
BG2814-6	*C. frutescens*	CMV	Partially resistant (incompletely resistance)	CMV-V26, CMV-V28, CMV-V27, CMV-NY, CMV-Fny and CMV-CA	–	Grube et al. (2000b)
LS 1839-2-4	*C. frutescens*	CMV	Resistant	CMV-Y.	–	Suzuki et al. (2003)
Tabasco	*C. frutescens*	CMV	Resistant	CMV-Y.	–	Suzuki et al. (2003)
Sapporo-oonaga	*C. annuum*	CMV	Resistant	CMV-Y.	–	Suzuki et al. (2003)
Nanbu-oonaga	*C. annuum*	CMV	Resistant	CMV-Y.	–	Suzuki et al. (2003)
MRCH	*C. annuum*	CMV	Resistant	CMV strain isolated from melon (Japan)	–	Monma and Sakata (1997)
Bukang	*C. annuum*	CMV	Completely resistant	CMV_Korean_ and CMV_FNY_	*Cmr1*	Kang et al. (2010)
BJ0747-1-3-1-1	*C. annuum*	CMV	Partially resistant	CMV-HB	*qcmv.hb-4.1* *qcmv.hb-8.2*	Yao et al. (2013)
I7339	*C. annuum*	CMV	Resistant	CMV-P1	*cmr3E cmr3L*	Min et al. (2014)
Lam32	*C. annuum*	CMV	Resistant	CMV-P1 CMV_Korean_ and CMV_FNY_	*cmr2*	Choi et al. (2018)
DH218	*C. annuum*	PVMV	Completely resistant	Isolates from Ghana and Cote d’Ivoire	*pvr6*	Caranta et al. (1996)
PBC688	*C. frutescens*	CMV	Incompletely resistance	CMV_FNY_	*qCmr2.1 (CA02g19570)*	Guo et al. (2017)
PI159234	*C. chinense*	PVY	Completely resistant	Pathotype (0,1)	*Pvr4+*	Arnedo-Andrés et al. (2002)
CM334	*C. annum*	PVY	Completely resistant	Pathotype (0), (1), (1,2)	*Pvr4*	Kim et al. (2015)
CV3 and CV8	*C. annuum*	ChiVMV	Resistant	Not specified	*Cvr1*	Lee et al. (2017)
CV9	*C. annuum*	ChiVMV	Resistant	Not specified	*cvr4*	Lee et al. (2017)
CV4	*C. annuum*	ChiVMV	Resistant	Not specified	*Cvr2-1+ Cvr2-2*	Lee et al. (2017)
NW4	*C. annuum*	ChiVMV	Resistant	ChiVMV strains from infected leaves of *Nicotiana benthamiana*	*Cvr1*	Lee et al. (2017)
IRH2451	*C. annuum*	ChiVMV	Resistant	*ChiVMV-_Bangalore_*	*-*	Ponnam et al. (2023)

*Cucumber Mosaic Virus*, PVY *and* ChiVMV resistance has been identified in various genetic sources of pepper (**Table 3**). However, host resistance in commercial varieties grown in Sub Saharan Africa, especially for CMV and PVMV is rare. There is a clear need to utilize the available and new genetic resources to move host resistance to these devastating viral diseases into consumer preferred backgrounds and develop and release multiple-virus resistant cultivars for African markets.

**Table 3:** Molecular markers linked to the genes resistant to PVMV, ChiVMV and PVY in pepper.

Virus	Resistance locus	Chr	Marker or gene	Type of marker	Population			Inheritance pattern	Status of research	Reference
					Parents	Generation	Number of plants			
**PVMV**	*pvr1=pvr2*	4	Pvr1-S, pvr1-R1, pvr1-R2	CAPS	R and S accessions	Line	23	Single recessive	Marker development	Yeam et al. (2005)
	*pvr1=pvr2*	4	eIF4E-A614G, -G325A, -T236G, -T200A	ARMS-PCR	‘Yolo Wonder’ × ‘CM334’, ‘Perennial’ × ‘Yolo Y’, ‘Perennial’ × ‘Florida VR2’	F2	–	Single recessive	Marker development	Rubio et al. (2008)
	*pvr1=pvr2*	4	KASP_pvr1	KASP	‘Habanero’ × ‘PI159234’	F2	56	Single recessive	Marker development	Holdsworth and Mazourek (2015)
	*pvr6*	3	eIF(iso)4E gene-based marker	InDel	‘DH218’ × ‘F’	F2	182	Single recessive	Gene cloned	Sandrine et al. (2005)
	*pvr6*	3	Pvr6-SCAR	SCAR	Dempsey’ × ‘Perennial	F2	187	Single recessive	Marker development	Hwang et al. (2009)
**ChiVMV**	*Cvr1*	6	BAC_Cvr1-1 BAC_Cvr1-3	SNP	CV3 x Jeju CV8 x Jeju	F2 and BC**_1_** F**_1_**	300	Monogenic dominant	Marker development	Lee et al. (2017)
**PVY**	*pvr2^2^*	4	T236G	SNP (ARMS-PCR)	Florida VR2 x Perennial	F2	16	recessive	Marker design	Rubio et al. (2008)
	*pvr2^2+^*	4	T200A	SNP (ARMS-PCR)	Yolo wonder x Perennial	F2	16	recessive	Marker design	Rubio et al. (2008)
	*pvr2^1^*	4	_G325A_	SNP(ARMS-PCR)	Perennial x Yolo Y	F2	16	recessive	Marker design	Rubio et al. (2008)
	*pvr2^3^*	4	_A614G_	SNP(ARMS-PCR)	Yolo wonder x Criollo de Morelos	F2	16	recessive	Marker design	Rubio et al. (2008)
	*Pvr4*	10	SCUBC19_1423_	SCAR	Serrano Criollo de Morelos-334 x Yolo Wonder	F2	110	Dominant	Marker development	Dogimont et al. (1996); Arnedo-Andrés et al. (2002)
	*pvr1^4^*	4	Pvr1-S, pvr1-R1, pvr1-R2	CAPS	SCM-334 x Yolo wonder	Line	–	Monogenic recessive	Marker design	Arnedo-Andrés et al. (2002)
	*Pn1*	4	Pvr1-S, pvr1-R1, pvr1-R2	CAPS	SCM-334 x Yolo wonder	Line	–	Monogenic recessive	Marker design	Arnedo-Andrés et al., (2006)

Adapted from (Barka and Lee, 2020).

## 7 Resistance genes and molecular markers for aphid-transmitted viruses

Several potyvirus resistance (*pvr*) genes have been reported in *Capsicum* species. The majority of *pvr* genes (*pvr1, pvr3, pvr5, pvr6, and pvr8*) are associated with a recessively inherited phenotype (Caranta et al., 1996; Kyle and Palloix, 1997; Grube et al., 2000a). The complementation between recessive *pvr6* (‘Perennial’) and *pvr2^2^* (‘Florida VR2’) genes have been reported to confer complete resistance to PVMV (Caranta et al., 1996). The *pvr6* gene was positioned on linkage group 4 (LG4) of a pepper map generated by using a DH population from the hybrid between ‘Perennial’ and ‘Yolo Wonder’ (Caranta et al., 1996) and was identified to correspond to an eIF(iso)4E gene, which encodes the second cap-binding isoform identified in plants (Sandrine et al., 2005). Two simultaneous recessive alleles at *pvr2* (eIF4E) and *pvr6* (eIFiso4E) loci were reported to confer resistance to PVMV as well as ChiVMV in pepper (Sandrine et al., 2005; Hwang et al., 2009). A dominant *Pvr4* gene for PVY resistance from *C. annuum* ‘CM334’ was located on pepper chromosome 10 (Dogimont et al., 1996; Grube et al., 2000a) and was mapped to a region containing eight AFLP markers; E33/M54-126, E41/M49-645, E38/M61- 403, -414, -460, E41/M55-102, E41/M49-296, and E41/M54-138, and one of them, E41/M49-645 was converted into a CAPS marker (Caranta et al., 1999), facilitating MAS.

RAPD and SCAR markers, UBC191432 and SCUBC191432, linked to the *Pvr4* locus were developed using segregating progenies obtained by crossing a homozygous resistant variety (Criollo de Morelos-334 (CM334)) with a homozygous susceptible variety (‘Yolo Wonder’) (Arnedo-Andrés et al., 2002). Arnedo-Andrés et al. (2002) and Dogimont et al. (1996) detected one monogenic dominant gene, *Pvr4* on chromosome 10, using an F_2_ population derived between crossing of CM334 and Yolo Wonder, conferring resistance to PVY. The gene was detected by Kim et al. (2017) using QTL mapping in an F_2_ population derived from by crossing CM334, Jupiter and ECW123R.

The variety Likeumjo was reported to be resistant to CMV-P0 (Kang et al., 2010) and resistance was found to be controlled by a single dominant gene, *Cucumber mosaic resistant* 1 (*Cmr1*), located on chromosome 2 and molecular markers linked to *Cmr1* have been developed (Kang et al., 2010). Several studies on CMV resistance in different *Capsicum* species have resulted in the general consensus that resistance is quantitatively controlled. Two additive QTLs and one epistatic QTL were identified using 94 DH lines obtained from the F1 derived from ‘Perennial’ crossed by ‘Yolo Wonder’ (Caranta and Palloix, 1996; Caranta et al., 1996). In the same population, a major QTL for CMV resistance was positioned on chromosome 12, with an R^2^ (coefficient of determination) of 19% and a strong linkage with the A5.1 marker (Pflieger et al., 2001).

## 8 Approaches to improve aphid-transmitted viruses resistance in pepper

### 8.1 Pathogens population and phylogenetics analysis

**Figure 5:**
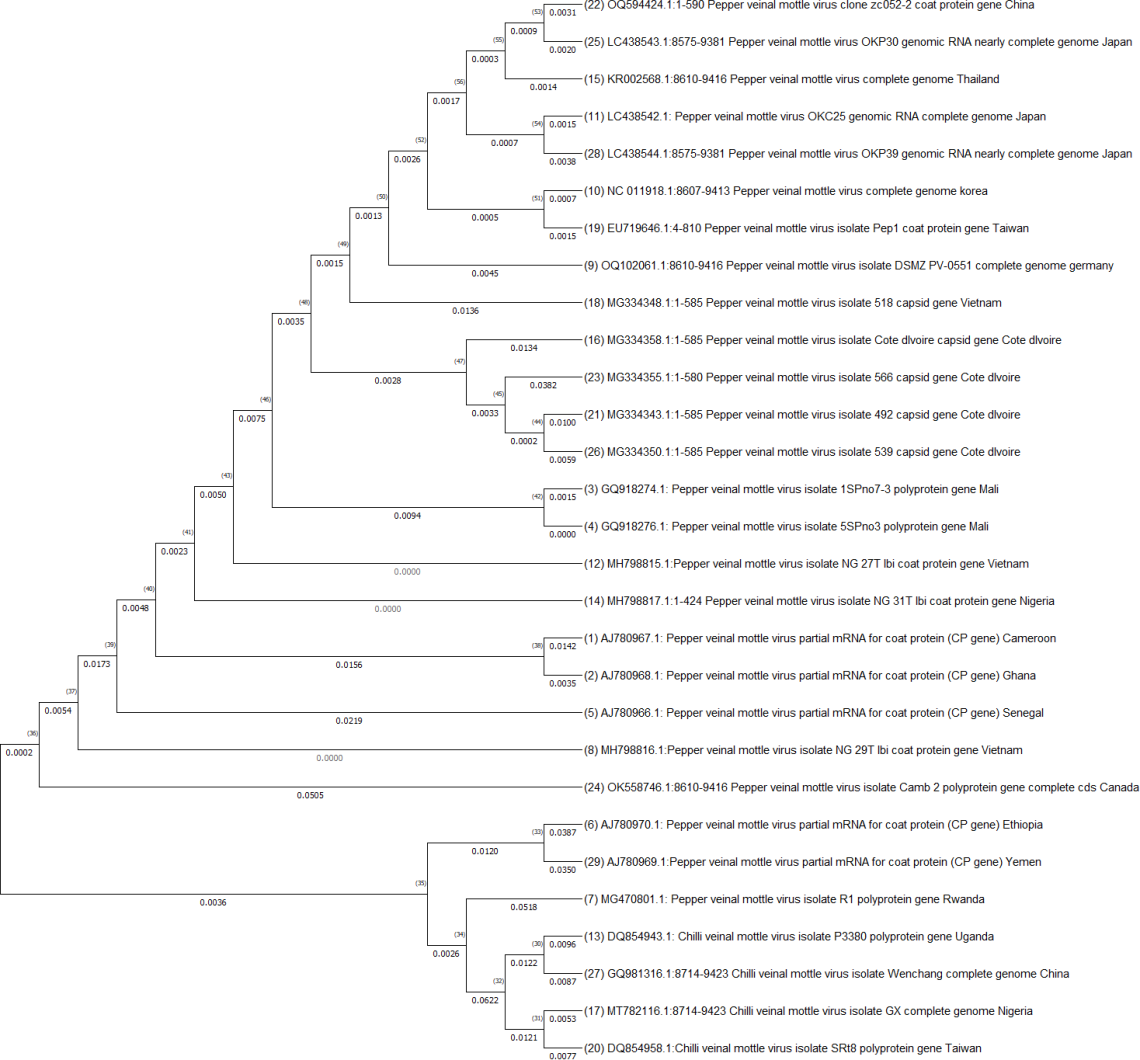
Phylogenetic analysis of PVMV viruses isolates partial genome sequences retrieved from NCBI GenBank. The sequences were aligned using MUSCLE and the tree constructed in MEGA by using Neighbor-Joining method following maximum likelihood criterion with 1000 bootstrap. The scale bar represents the rate of nucleotide substitutions per site.

Effective management of plant viral diseases hinges upon a comprehensive understanding of the pathogen populations in major production areas. To gain a deeper insight into these populations, diversity analyses of the different virus strains must be conducted. In the case of aphid-transmitted pepper viruses (*Potyvirus, Cucumovirus*, and *Polerovirus*), a thorough grasp of their strain diversity and molecular properties is crucial for developing adequate disease management strategies. Several research efforts have been undertaken to identify and characterize strains of PVMV, CMV, PVY, ChiVMV, and PeVYV in Africa. Genetic and evolutionary studies have proven to be valuable tools for elucidating the molecular basis of virus geographical spread, adaptation to new hosts, and designing more effective epidemic control strategies (Elena et al., 2011). To assess the genetic diversity of PVMV, the partial genome sequences of 29 isolates of PVMV were retrieved from the NCBI GenBank and subjected to phylogenetic analysis (**Figure 5**). The African isolates from countries like Cameroon (AJ78067.1), Ghana (AJ780968.1), Mali (GQ918274.1 and GQ918276.1), Senegal (AJ780966.1), Rwanda (MG470801.1) and Nigeria (MH798817.1 and MH798816.1) were grouped together, which indicates they share a close evolutionary history and likely have a common geographic origin. Isolates from Cameroon, Ghana and Senegal form a close group, suggesting they have a recent common ancestor. The isolate from Mali displayed in a separate subgroup, and those from Rwanda and Nigeria in different subgroups, indicating significant divergence. Isolates from non-African regions such as China, Japan and Thailand formed separate clusters, support genetic divergence from African isolates. Importantly no African isolate was genetically identical to non-African isolates, highlighting that PVMV evolution has been independent on these two continents. Our analysis is aligned with the results of Laina et al. (2019), who reported that African isolates of PVMV exhibit substantial genetic diversity, which can pose challenges for breeding efforts for host resistance. Consequently, future pepper breeding initiatives for host resistance should take into account isolate variability to effectively target moving strain-specific resistance genes into African pepper for better control and management of aphid-transmitted viruses.

Diversity among CMV isolates has also been observed from different African countries (Waweru et al., 2019; Apalowo et al., 2022), but there is a lack of research focused on aligning the genome sequences of isolates from distinct African countries to assess their relationship and diversity. In light of this, it would be prudent to prioritize future research efforts on understanding isolate diversity and conducting relationship analysis before embarking on extensive host resistance breeding programs in Africa.

As reported by Tomassoli et al. (2016), African PeVYV strains cluster into two distinct clades, with West African (Mali and Ivory Coast) forming one clade, which differs from the clade containing the Sudan isolate. Significant diversity among PeVYV isolates from various African countries have been reported (Afouda et al., 2017; Waweru et al., 2019). Therefore, it is essential to establish a phylogeny tree of existing PeVYV isolates from each African country based on their diversity and prevalence before developing comprehensive breeding programs for better disease management.

### 8.2 Diversity sets and validation of host resistance

**Figure 6:**
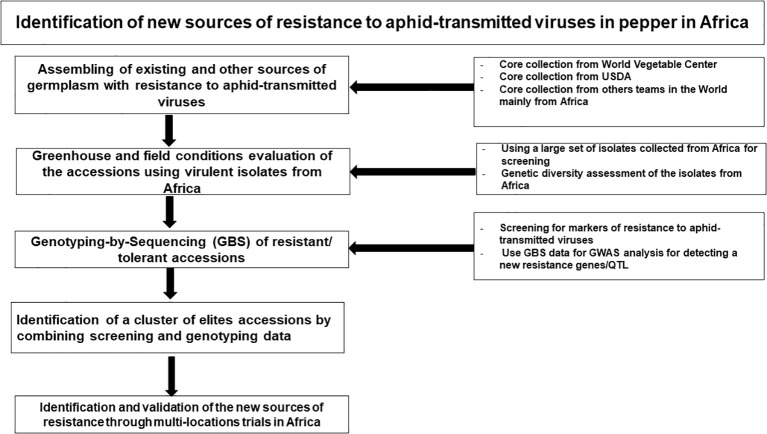
Identification pathway for new sources of resistance to aphid-transmitted viruses in pepper in Africa.

Field screening is a cost-effective and relatively straightforward technique for identifying sources of host resistance to viruses, but it can lead to mixed infections from non-target viral species and genera, making it challenging to accurately identify resistance sources (Kenyon et al., 2014; Jo et al., 2017; Nalla et al., 2023). To address this challenge, we propose developing a collection of pepper diversity sets with known resistance genes to the various aphid-transmitted viruses, based on core collections, breeding lines, and germplasm accessions with reported field tolerance to the important viral diseases in Africa (**Figure 6**). This diversity set can be screened under field conditions in the major pepper-growing regions in Africa, where the target viruses are prevalent. Based on the resistance response of the diversity set, in combination with diagnostics, single and mixed-infection resistance can be identified, providing a foundation for future research (**Figure 6**). The most resistant accessions will then be selected for greenhouse screening by mechanical inoculating with the most predominant and most severe viral strains in single and mixed infections to determine whether they harbor resistance genes for single and mixed infection, as described by Suzuki et al. (2003), who screened pepper accessions for resistance to CMV, *Tomato aspermy virus* (TAV), *Tomato mosaic virus* (ToMV), *Pepper mild mottle virus* (PMMoV), and *Tomato spotted wilt virus* (TSWV) under field and greenhouse conditions. Subsequently, molecular markers for resistance genes and virus presence will be employed to validate the field-observed virus resistance status of the accessions. If resistant accessions lack known resistant genes but are symptom-free in the field and under artificial inoculation, further investigation would be needed to elucidate the mechanism underlying resistance beyond known resistant genes.

If the diversity set for resistance proves ineffective, then it would be required to utilize the global *Capsicum* core collection (McLeod et al., 2023) and inoculate using the predominant strains individually. Likely, the core collection will result in the identification of mostly moderate levels of resistance, and SNP associated with host resistance can be mapped using genome wide association studies (GWAS). Pyramiding of multiple genes associated with moderate resistance, based on the SNPs found using GWAS in the core collection into a single background can result in higher levels of resistance. The highly resistant lines developed from gene pyramiding can then be deployed in breeding programs and for further validation experiments.

### 8.3 Host resistance against mixed infection and marker-assisted gene pyramiding

Pyramiding resistance genes involve combining multiple resistance genes into a single plant to enhance and broaden resistance. This can be done through conventional breeding or using molecular tools to stack genes that confer resistance to different strains or species of potyviruses. The breakdown of plant virus resistance genes is a major issue in agriculture. Djidjou-Demasse et al. (2017) investigated whether a set of resistance genes would last longer when stacked into a single plant cultivar (pyramiding) or when deployed individually in regional mosaics (mosaic strategy). Mosaics are more versatile than pyramiding strategies, and we found that deploying a mosaic of three to five resistance genes generally provided effective disease control, unless the epidemics were driven mostly by within-field infections. Djidjou-Demasse et al. (2017) found that pyramiding strategies performed better only with slowly changing virus reservoir dynamics. It is known that gene pyramiding is not going to completely solve the problem, because of the synergistic interaction of different viruses and strains. Combined host resistance to the individual viruses in a single background could still have symptoms in the presence of multiple viruses. There is a need to explore the possibility of developing host resistance in the presence of mixed infections.

**Figure 7:**
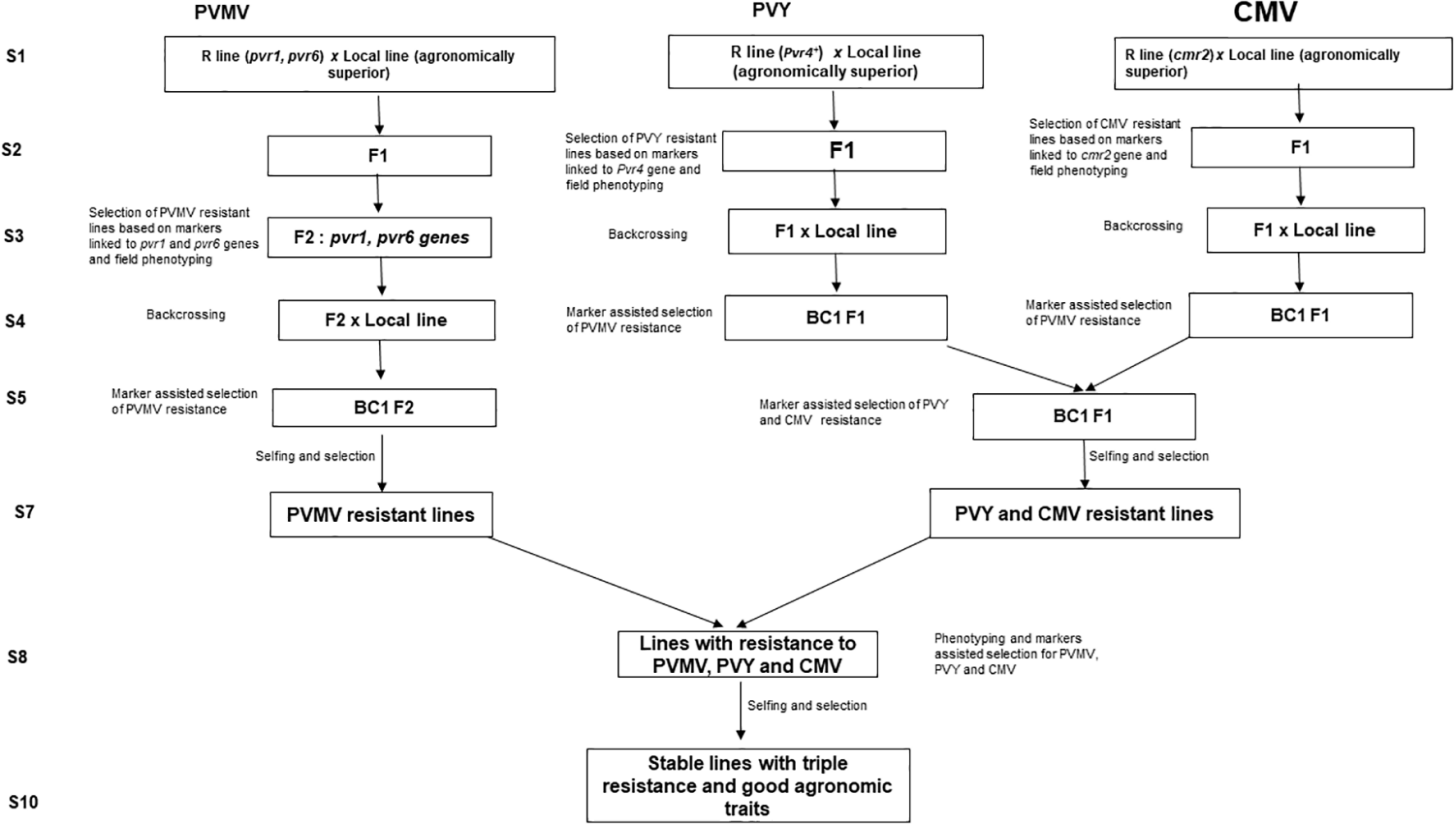
Breeding plan for developing aphid-transmitted virus resistant lines using pyramiding marker assisted selection.

The application of marker-assisted selection (MAS) has facilitated breeding for crop improvement, especially for phenotype traits controlled by quantitative trait loci or recessive allele (Li et al., 2020). Marker-assisted selection has been successfully used in efficient selection of many resistance genes in pepper crop improvement (Ridzuan, 2018). Alternatively, several modern breeding strategies, such as, marker-assisted backcrossing (MABC), marker-assisted recurrent selection (MARS), and marker-assisted pedigree selection (MAPS), have been also used for resistant breeding in pepper (Li et al., 2020). For aphid- transmitted resistance breeding, susceptible and resistant genotypes can be precisely identified using molecular markers at an early stage of plant growth, without requiring field screening with artificial inoculation or any environmental influence for most of the viruses. So far there are developed markers for PVMV, PVY and CMV but no markers are available for PeVYV yet. We still need to rely on artificial inoculation to evaluate a germplasm for resistance to PeVYV. The **Figure 7** proposed a breeding plan using pyramiding marker assisted selection. The most stable PVMV resistant line reported and tested is DH801 with *pvr1* and *pvr6* recessive genes (Moury et al., 2005). PI159234 contains *Pvr4* which confers resistance to PVY (Dogimont et al., 1996) and LAM32 with *cmr2* can confer high resistance to CMV (Choi et al., 2018). Those lines can be used here as resistant sources to improve susceptible cultivar with superior agronomic traits. Pyramiding genes techniques will be used and can be fixed more than 10 seasons of crossing and selfings. Markers developed for these viruses will be used to fast-track resistance genes in the population every season (**Figure 7**).

### 8.4 Genome wide association studies

GWAS identifies genetic variations associated with resistance by scanning the genomes of many different plant lines. This information can pinpoint candidate genes or loci responsible for resistance, aiding in the development of resistant varieties. GWAS have become a powerful tool for dissecting the complex genetic architecture of plant virus resistance. This approach identifies associations between specific DNA markers (SNPs) across the entire genome and a plant’s susceptibility or resistance to a particular virus (Tam et al., 2019). GWAS has led to the discovery of novel genes involved in plant’s defense mechanisms against viruses (Tam et al., 2019). Beyond identifying individual resistance genes (Quantitative Trait Loci, QTLs), GWAS can also detect interactions between these genes, providing a more comprehensive picture of the genetic basis for resistance. Numerous successful applications of GWAS have been conducted in crops like soybean and maize. These studies have uncovered QTLs associated with resistance to viruses such as Soybean Mosaic Virus (SMV) and Tobacco Ringspot Virus (TRSV) in soybean (Chang et al., 2016; Liu et al., 2019; Che et al., 2020; Chu et al., 2021; Pu et al., 2024), and *Maize Chlorotic Mottle Virus* (MCMV) resistance in maize (Sitonik et al., 2019). Additionally, GWAS can help identify genes with minor effects on resistance, which can be combined with major resistance genes to create more durable resistance (Pilet-Nayel et al., 2017). For example, Tamisier et al. (2020) employed GWAS to investigate the genetic factors controlling PVY infection levels in pepper. Their analysis of over 260 pepper accessions identified seven SNPs significantly associated with resistance, located on chromosomes 4, 6, 9, and 12. Notably, two SNPs on chromosome 4 mapped near the *pvr2* gene, encoding the eukaryotic initiation factor 4E (eIF4E), which is known to play a role in plant defense. Interestingly, SNPs on chromosomes 6 and 12 colocalized with previously reported QTLs for PVY resistance. Recent advancements in Next-Generation Sequencing (NGS) technologies have further enhanced the power of GWAS in plant virus resistance studies. Eun et al. (2016) utilized Genotyping-by-Sequencing (GBS) to identify two novel major QTLs associated with resistance to *Pepper mottle virus* isolate P1 (CMV-P1) in pepper. Another example is Tamisier et al. (2022), who utilized double-digest restriction associated DNA sequencing (ddRADseq) to pinpoint genetic markers linked to PVY tolerance on chromosome 9 in pepper.

### 8.5 Genomic Selection

Genomic Selection (GS) uses genome-wide markers to predict the breeding values of plants for complex traits like disease resistance. By creating predictive models based on genotypic and phenotypic data, breeders can select plants with the highest potential for resistance, thus accelerating the breeding process. Genomic selection can be used to select superior progenies or genotypes from large germplasm sets without needing phenotypic evaluation. A genomic prediction model was developed to accurately predict sensitivity of soybeans (*Glycine max* (L.) Merr.) to *Tobacco ringspot virus* (TRSV; *Nepovirus*) with a correlation of R = 0.67 (*P* < 0.01) (Chang et al., 2016). Chang et al. (2016) concluded that the genomic prediction model is a promising tool for identifying soybeans that are resistant to TRSV. Similarly, GS can be deployed in pepper to predict resistance or susceptibility of pepper accessions to aphid-transmitted virus in Africa. However, this would need to be done for resistance to a single virus species at a time, and not for all aphid-transmitted viruses at once.

### 8.6 Gene editing

CRISPR/Cas9 gene editing allows for precise editing of the plant genome to either knock out susceptibility genes and can be deployed to develop chili with targeted improvements in virus resistance without introducing foreign DNA, which is advantageous for regulatory approval and consumer acceptance. Gene editing provides a significant opportunity for resistance to potyviruses, which are often reported to be controlled by recessive genes. Plant genes encoding the eukaryotic translation initiation factors (eIF) represent promising targets for engineering viral resistance using new plant breeding techniques (Bastet et al., 2018). In Solanaceae two variants of eIF4E gene, eIF4E1 and eIF4E2, with 70-80% homology and eIF(iso)4E with 50% homology to eIF4E1/eIF4E2 were identified and PVY resistant transgenics were developed via overexpression of eIF4E gene variant (Gutierrez Sanchez et al., 2020). Yoon et al. (2020) used CRISPR/Cas9 gene editing to introduce mutations in the eIF4E1 gene of tomato plants. Transgenic tomato plants that expressed the Cas9 protein and a guide RNA targeting the eIF4E1 gene were screened for individuals that had mutations in the eIF4E1 gene. They found that some of these plants had mutations that resulted in the deletion of 11 to 43 base pairs of DNA in the eIF4E1 gene. The mutated eIF4E1 gene was then transferred into non-transgenic tomato plants, which were found to be inherited by their offspring. The mutant tomato plants were resistant to PepMoV but not TEV. Therefore, CRISPR/Cas9 gene editing has potential to be used to introduce mutations in the eIF4E1 gene that confer resistance to the potyviruses that infect pepper in Africa.

### 8.7 RNAi technology

RNA interference (RNAi) offers a powerful strategy for engineering plant resistance against viruses. RNAi works by silencing specific genes within the virus itself, hindering its ability to replicate or cause symptoms in the plant (Taliansky et al., 2021). The effectiveness of RNAi for plant disease resistance has been successfully demonstrated in various studies. Hameed et al. (2018) engineered potato plants with near-complete resistance to three potyviruses using a single chimeric hairpin cassette targeting viral coat protein sequences. Similarly, Ntui et al. (2014) constructed an RNAi tool targeting the CMV replicase gene in tomato plants, which displayed continued immunity or high resistance even when challenged with a closely related CMV strain, highlighting the broad-spectrum potential of RNAi. Notably, no viral presence was found in the resistant plants, further supporting the link between RNAi-mediated silencing and enhanced disease resistance. The RNAi approach is not limited to a single virus and transgenic pepper plants engineered to express CMV-specific siRNAs exhibited delayed symptom development and significantly reduced disease severity upon infection with various CMV strains (Li et al., 2020). Additionally, introducing defense-related genes involved in the jasmonic acid (JA) signaling and antiviral RNA silencing pathways represents another potential strategy for regulating plant resistance against viruses (Li et al., 2020; Yang et al., 2020).

### 8.8 Gene silencing

Innovations such as virus-induced gene silencing (VIGS) and artificial microRNAs (amiRNAs) offer new ways to study gene function and develop resistant plants by transiently or stably silencing virus-related genes. Gene silencing is a powerful tool that can be used to downregulate the expression of a gene at either the transcriptional or post-transcriptional level. Transcriptional gene silencing occurs when the process of transcription is prevented, while post-transcriptional gene silencing occurs when mRNA is degraded (Sharma et al., 2021). In addition to small RNA molecules, viruses can also carry out gene silencing, which is known as virus-induced gene silencing (VIGS). VIGS involves cloning and inserting endogenous gene sequences into recombinant viral vectors that are then inoculated into plants (Baulcombe, 1999), which triggers RNA-mediated gene silencing. VIGS is a technology that can be used to repress the expression of a gene of interest in plants and does not require sequence information, making it a more versatile tool than other gene silencing techniques (Shreya et al., 2019). Gene silencing has been used as a reverse genetic tool to develop resistance to various biotic and abiotic stresses, as well as to improve yield and quality parameters (Bekele et al., 2019).

### 8.9 Targeting-Induced Local Lesions IN Genome

The advent of new technologies, such as Targeting-Induced Local Lesions IN Genome (TILLING), has opened up a new era for the development of genetic resistance in crops (Hofinger et al., 2009; Piron et al., 2010). These technologies allow the identification of target gene mutants from an artificially induced mutation population. For instance, TILLING was employed to screen for eIF4E or eIF4G mutants in an ethyl methanesulfonate (EMS)-induced tomato mutant population (Piron et al., 2010). A splicing mutant Sl-eIF4E1 (G1485A) was identified to be immune to PVY and PepMoV. The mutated gene Sl-eIF4E1 (G1485A) encodes a truncated protein that is impaired in cap-binding activity (Piron et al., 2010). A TILLING-based method to identify natural nucleotide diversity, termed EcoTILLING, has also been employed successfully to identify allelic variants of eIF4E against MNSV in melon (Nieto et al., 2007) and against PVY in pepper (Ibiza et al., 2010). This approach may be particularly effective for heterozygous species in which recessive alleles may exist, but cannot be screened out by the conventional phenotypic resistance assay.

### 8.10 Mutation breeding

Mutation breeding, such as the use of ethyl methanesulfonate (EMS) or gamma ray, can be used to induce novel mutations and result in the development of recessive alleles of susceptibility genes, resulting in resistance. EMS mutation has been deployed to develop new mutations and resulted in host resistance to ChiVMV in pepper (Siddique et al., 2020). Mutagenesis, particularly through EMS, has become a valuable tool for researchers to understand plant disease resistance mechanisms. By inducing mutations in genes, scientists can observe the resulting effects and identify those involved in defense (Abe et al., 2012). Within the Solanaceae family encompassing tomato, eggplant, and pepper, EMS mutagenesis has proven particularly successful. Studies have demonstrated its effectiveness in generating a wider range of morphological traits and enhancing desirable characteristics such as yield, fruit quality, disease resistance, and even male sterility (Piron et al., 2010; Gauffier et al., 2016; Xi-Ou et al., 2017). For instance, EMS-induced mutations in the eukaryotic initiation factor 4E (eIF4E) gene in tomato conferred resistance to potyviruses (Piron et al., 2010; Gauffier et al., 2016). Similarly, EMS mutagenesis in eggplant resulted in alleles increased levels of beneficial phenolic compounds (Xi-Ou et al., 2017). In pepper, numerous studies have utilized mutant populations derived from EMS mutagenesis (Bosland, 2002; Jabeen and Mirza, 2002; Hwang et al., 2014; Siddique et al., 2020). One example is an EMS mutant population of the sweet pepper cultivar Maor, developed to investigate genes controlling flower and plant architecture (Jo and Kim, 2019). For chili peppers, recessive resistance to potyviruses is often mediated by mutations in eIF genes. These mutations disrupt the interaction between eIF genes (e.g., *pvr1* or *pvr6*) and the viral VPg protein, hindering viral replication (Sanfaçon, 2015). This concept is exemplified by the double mutation in *pvr1* (eIF4E) and *pvr6* (eIFiso4E) that confers resistance to ChiVMV (Hwang et al., 2009) and more recently, a key gene for *Begomovirus* resistance in sweet peppers (Koeda et al., 2021). Additionally, through EMS mutagenesis 15 *Pepper yellow leaf curl virus* (PepYLCV; *Begomovirus*)-resistant mutant lines from Gelora cultivar were identified (Manzila and Priyatno, 2020). While EMS mutagenesis offers compelling evidence for virus resistance discovery, gamma radiation, which induces a broader spectrum of mutations at a lower rate, warrants further exploration.

### 8.11 Other management strategies of aphid-transmitted viruses in pepper

**Figure 8:**
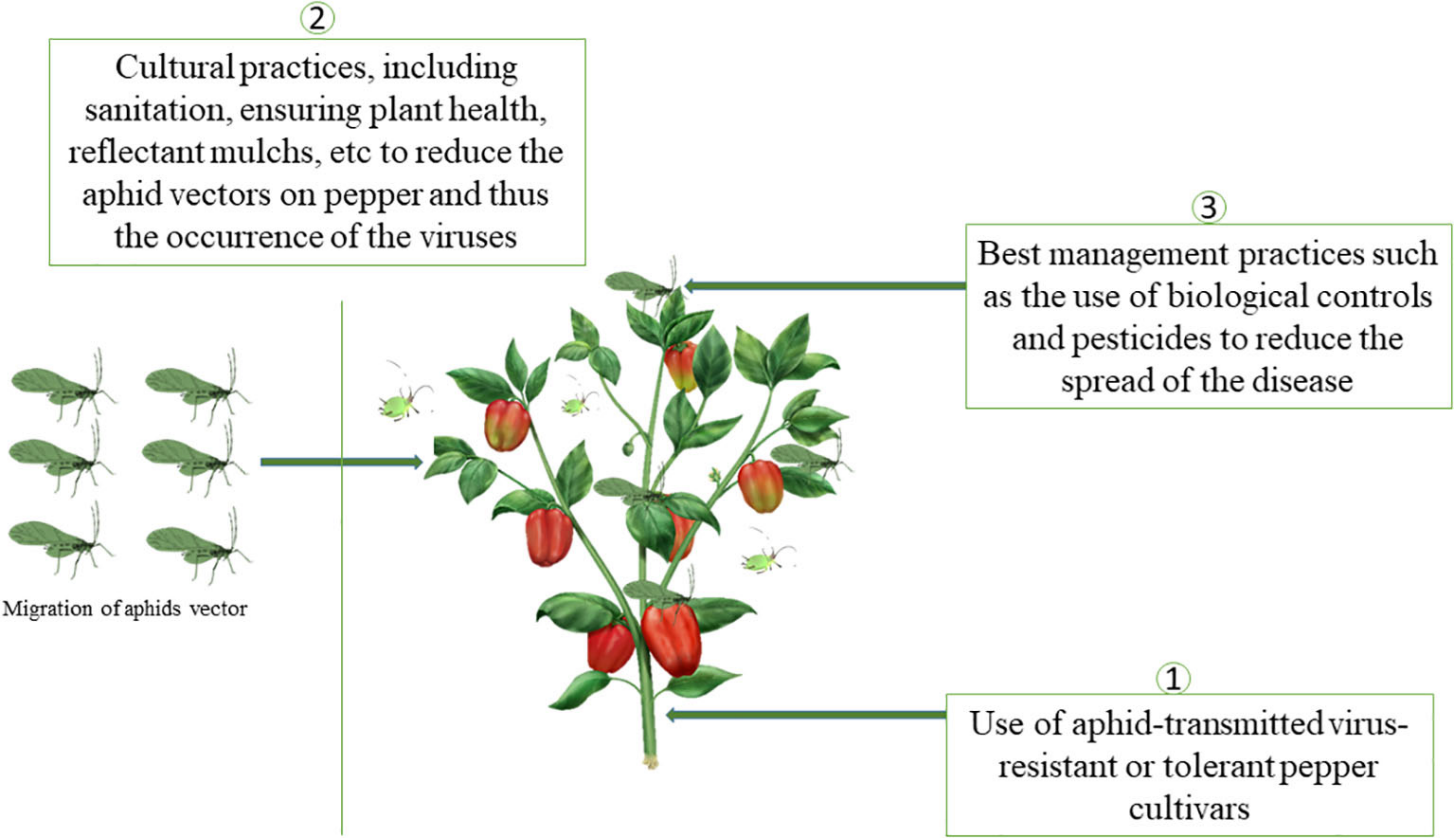
Sustainable and effective management methods against aphids- transmitted viruses in pepper.

Management strategies have been developed and are recommended for the effective management of aphid-transmitted viruses in pepper. Three promising options proposed included (1) using resistant pepper varieties, (2) implementing cultural practices to reduce the arrival of aphid’s vectors and (3) the use of chemical and biological pesticides to reduce the spread of the disease (**Figure 8**). The combination of strategies is likely the most effective way to reduce losses associated with aphid-transmitted viruses. Here, we will discuss more about the second and third management options.

#### 8.11.1 Cultural practices

Cultural practices to control aphid-transmitted viruses include preventing or reducing the occurrence of the disease in the field and reducing the spread of the disease when it is detected (Hooks and Fereres, 2006). More importantly, maintaining spatial separation between fields to reduce the transmission risk from nearby infected crops is a common cultural practice for managing vector-borne diseases (Jones, 2001). Also, alternative hosts, such as weeds in neighboring fields are an important potential source of virus, as they can serve as a reservoir for the vector, between seasons. For instance *Chromolaena odorata*, *Ageratum conyzoides*, *Boerhavia diffusa*, *Croton hirtus*, *Euphorbia heterophylla*, *Centrosema pubescens* and *Solanum torvum* have been reported as a reservoir for PVY-n (Potato virus Y-necrotic); PVMV and CMV (Traore et al., 2013; Kouadio et al., 2015). During pepper production, it is important to implement appropriate strategies to protect the crop. Effective management strategies include disease-free seeds of resistant varieties, and produce seedlings in protected environments (like insect-proof nets or greenhouses) to minimize vector contact and initial virus infection (Dáder et al., 2015). The use of aluminum foil or reflective plastic mulch has been reported to reduce aphid populations (Loebenstein et al., 1975). It has been shown that the association of pepper (in border or intercropping) with large crops, such as plantain, cassava, sorghum or maize, help to reduce aphids populations and movement as well as the incidence of viral diseases (Emden and Harrington, 2017).

#### 8.11.2 Biological control

Biological control is also an important control method based on the use of predators, parasitoids, pathogens, antagonist or competitor population, without using pesticides (Stenberg et al., 2021). The goal of biological control is to maintain the populations of bio-aggressors below a threshold of harmfulness (Stenberg et al., 2021). The main predators of aphids are ladybird beetles (Coleoptera: Coccinellidae), with *Cheilomenes* spp. being the most commonly used for management of aphids (Kawakami et al., 2016). Two species of predators were identified by Sæthre et al. (2011) in Benin, *Cheilomenes propinqua* (Mulsant, 1850) and *Cheilomenes sulphurea* (Olivier, 1791). Lacewings (Chrysopidae and Hemerobiidae), Nabid and mirid bugs (Nabidae and Miridae) are other predators of aphids (Regnault-Roger, 2020). Parasitoids of aphids have been found and evaluated, with members of Aphidiinae representing the most commonly used parasitoids, which belong to the monophyletic subfamily of Braconidae (Hymenoptera). The members of Aphelinidae (Hymenoptera) and Cecidomyiidae are also specialized parasitoids of aphids (Boivin et al., 2012). The aphid parasitoids *Lysiphlebus testaceipes* Cresson (Hymenoptera: Braconidae, Aphidiinae) and *Aphelinus* sp. Dalman (Hymenoptera: Aphelinidae) were identified by (Sæthre et al., 2011) to effectively control *Aphis gossypii* infesting pepper plants (Kamel, 2011; Tepa-Yotto et al., 2013).

#### 8.11.3 Chemical control

Current control strategies for aphids regularly include pesticide applications (Hooks and Fereres, 2006). Farmers typically rely on the excess and indiscriminate use of chemical pesticides to control vectors in Sub-Saharan Africa (Biao and Afouda, 2019; Waweru et al., 2020b; Zohoungbogbo et al., 2024). With the growing concern about the environmental impact of insecticide abuse, the use of biological pesticides is increasingly being recommended to control aphids. For example, (Biao et al., 2018) demonstrated that aqueous extracts of garlic, hyptis leaves and neem seed could be an alternative to synthetic pesticides for integrated management of pepper virus diseases and their vectors.

## 9 Conclusion

Aphid-transmitted viruses are a significant threat to pepper production in Africa. Developing sustainable and effective control measures to mitigate yield losses due to the aphid-transmitted virus is crucial. Developing varieties that resist the major aphid-transmitted viruses (PVMV, CMV, PVY and PeVYV) is important. Although resistance sources against these aphids transmitted viruses are available, they have not been really tested in the regions of interest in Africa. Breeding for broad resistance against multiple viruses remains a challenge in Africa where mixed infection is more common than single infection. As a way forward, there is a need to identify germplasm resources with resistance against various aphid-transmitted virus strains, and subsequent pyramiding of the resistance using MAS could be an effective strategy. Pyramiding genes techniques can be used and the resistance can be fixed after more than 10 seasons of crossing and selfings for the three majors viral diseases identified (PVMV, CMV and PVY). Markers developed for these viruses will be used to fast-track resistance genes in the population every season with the possibility to develop stale triple resistant cultivars with good agronomic traits. In addition, MAS and GS can be used together to enhance selection accuracy, while CRISPR/Cas9 can be employed alongside traditional breeding to stack resistance genes. This integrative approach maximizes the strengths of each method and accelerates the development of aphid-transmitted virus resistance in pepper. The use of integrated pest management techniques that incorporate genetic resistance, cultural practices, and chemical control should be prioritized. Advances in molecular biology, genomics, transcriptomics, and bioinformatics can provide new insights into the mechanisms of host-pathogen-vector interactions and hasten the development of effective and long-term management strategies for aphid-transmitted viruses. The most effective breeding programs often combine several of these techniques.
