# Supplementary material for: Current knowledge and breeding strategies for management of aphid-transmitted viruses of pepper (Capsicum spp.) in Africa
Source: Front Plant Sci. 2024 Oct 25;15:1449889. doi: 10.3389/fpls.2024.1449889 (PMC11543480; doi:10.3389/fpls.2024.1449889)
Supplement: Supplementary file 1 [file Table1.docx]

Supplementary Material

# Supplementary Table 1. Citations of the reports of aphid-transmitted viruses in pepper in Africa.

| **Country** | **Aphid-transmitted viruses reported** | **Genus** | **Year of the first report** | **References** |
| --- | --- | --- | --- | --- |
| Benin | PVMV | Potyvirus | 2013 | Afouda, L. A., Kotchofa, R., Sare, R., Zinsou, V., & Winter, S. (2013). Occurrence and distribution of viruses infecting tomato and pepper in Alibori in northern Benin. *Phytoparasitica*, *41*, 271-276. |
|  | PVY | Potyvirus | 2013 | Afouda, L. A., Kotchofa, R., Sare, R., Zinsou, V., & Winter, S. (2013). Occurrence and distribution of viruses infecting tomato and pepper in Alibori in northern Benin. *Phytoparasitica*, *41*, 271-276. |
|  | ChiVMV | Potyvirus | 2022 | Zohoungbogbo, H. P., Achigan-Dako, E. G., Honfoga, J., Lin, S. W., Lin, T. H., Wang, Y. W., ... & Barchenger, D. W. (2022). Incidence and severity of aphid-transmitted viruses and horticultural performance of habanero pepper (Capsicum chinense Jacq.) breeding lines in Benin. *HortScience*, *57*(5), 606-612. |
|  | CMV | Cucumovirus | 2013 | Afouda, L. A., Kotchofa, R., Sare, R., Zinsou, V., & Winter, S. (2013). Occurrence and distribution of viruses infecting tomato and pepper in Alibori in northern Benin. *Phytoparasitica*, *41*, 271-276. |
|  | PeVYV | Polerovirus | 2017 | Afouda, L., Kone, D., Zinsou, V., Dossou, L., Kenyon, L., Winter, S., & Knierim, D. (2017). Virus surveys of Capsicum spp. in the Republic of Benin reveal the prevalence of pepper vein yellows virus and the identification of a previously uncharacterised polerovirus species. *Archives of virology*, *162*, 1599-1607. |
| Ghana | PVMV | Potyvirus | 1971 | Brunt, A. A., & Kenten, R. H. (1971). Pepper veinal mottle virus‐a new member of the potato virus Y group from peppers (Capsicum annuum L. and C. frutescens L.) in Ghana. *Annals of Applied Biology*, *69*(3), 235-243. |
|  | CMV | Cucumovirus | 2014 | Appiah, A. S., Quartey, E. K., Amoatey, H. M., Nunekpeku, W., Owusu-Ansah, M., & Ofori, S. (2014). Response of nine cultivars of pepper (Capsicum spp.) to infection by four viruses under natural field conditions in the coastal savanna zone of Ghana. *Research Journal of Applied Sciences, Engineering and Technology*, *7*(5), 903-907. |
| Mali | PVMV | Potyvirus | 2010 | Tsai, W. S., Abdourhamane, I. K., & Kenyon, L. (2010). First report of pepper veinal mottle virus associated with mosaic and mottle diseases of tomato and pepper in Mali. *Plant Disease*, *94*(3), 378-378. |
|  | PeVYV | Polerovirus | 2013 | Knierim, D., Tsai, W. S., & Kenyon, L. (2013). Analysis of sequences from field samples reveals the presence of the recently described pepper vein yellows virus (genus Polerovirus) in six additional countries. *Archives of virology*, *158*, 1337-1341. |
| Burkina-Faso | PVMV |  | 1996 | Huguenot, C., Furneaux M., Clare J.,Hamilton R. (1996). Serodiagnosis of Pepper Veinal Mottle Virus in West Africa using Specific Monoclonal Antibodies in DAS‐ELISA. Journal of Phytopathology 144, 29-32. |
| Nigeria | PVMV |  | 1975 | Lana, A. O., Gilmer, R. M., Wilson, G. F., & Shoyinka, S. A. (1975). An unusual new virus, possibly of the potyvirus group, from pepper in Nigeria. *Phytopathology*, *65*, 1329-1332. |
|  | PVY |  | 1975 | Lana, Allan O., et al. "An unusual new virus, possibly of the potyvirus group, from pepper in Nigeria." *Phytopathology* 65 (1975): 1329-1332. |
|  | CMV |  | 2012 | Arogundade, O., Balogun, O. S., & Kareem, K. T. (2012). Occurrence and distribution of pepper veinal mottle virus and cucumber mosaic virus in pepper in Ibadan, Nigeria. *Virology Journal*, *9*, 1-4. |
| Cote d’Ivoire | PVMV |  |  | De Wijs, J.-J. (1973). Pepper veinal mottle virus in Ivory Coast. Netherlands journal of plant pathology 79, 189-193. |
|  | CMV |  |  | Fauquet C, Thouvenel JC, 1987. Maladies virales des plantes en Côte d'Ivoire. Documentations Techniques 46. Paris, France: ORSTOM. |
|  | PeVYV |  |  | Bolou Bi, B. A., Moury, B., Abo, K., Kakou, D. J., Girardot, G., Kouassi, N. D. P., ... & Kone, D. (2015). First report of Pepper vein yellows virus in field-grown pepper in Ivory Coast. |
| Senegal | PVMV |  | 1996 | Huguenot, C., Furneaux M., Clare J.,Hamilton R. (1996). Serodiagnosis of Pepper Veinal Mottle Virus in West Africa using Specific Monoclonal Antibodies in DAS‐ELISA. Journal of Phytopathology 144, 29-32. |
|  | ChiVMV |  | 2005 | Moury, B., Palloix A., Caranta C., Gognalons P., Souche S., Selassie K. G.,Marchoux G. (2005). Serological, molecular, and pathotype diversity of Pepper veinal mottle virus and Chili veinal mottle virus. Phytopathology 95, 227-232. |
|  | PVY |  | 2001 | Moury, B., Palloix A., Caranta C., Gognalons P., Souche S., Selassie K. G.,Marchoux G. (2005). Serological, molecular, and pathotype diversity of Pepper veinal mottle virus and Chili veinal mottle virus. Phytopathology 95, 227-232. |
| Togo | PVMV |  | 1996 | Huguenot, C., Furneaux M., Clare J.,Hamilton R. (1996). Serodiagnosis of Pepper Veinal Mottle Virus in West Africa using Specific Monoclonal Antibodies in DAS‐ELISA. Journal of Phytopathology 144, 29-32. |
| Sierra-leone | PVMV |  | 1996 | Huguenot, C., Furneaux M., Clare J.,Hamilton R. (1996). Serodiagnosis of Pepper Veinal Mottle Virus in West Africa using Specific Monoclonal Antibodies in DAS‐ELISA. Journal of Phytopathology 144, 29-32. |
| Cameroon | PVMV |  | 1993 | Nono-Womdim, R. (2003). An overview of major virus diseases of vegetable crops in Africa and some aspects of their control. In *Plant Virology in Sub-Saharan Africa, Proc. Conf. Organized by IITA. J. d’A. Hughes and J. Odu, International Institute of Tropical Agriculture, Ibadan, Nigeria* (pp. 213-232). |
|  | CMV |  | 2022 | Deloko, D. C. T., Chofong, N. G., Ali, I. M., Kachiwouo, I. G., Songolo, F. O., Manock, A. R. N., ... & Njukeng, A. P. (2022). Detection of Cucumber mosaic virus on Solanum lycopersicum L. and Capsicum annuum L. in the Western region of Cameroon. *Journal of Agriculture and Food Research*, *8*, 100294. |
| Rwanda | PVMV |  | 2018 | Skelton, A., Uzayisenga, B., Fowkes, A., Adams, I., Buxton-Kirk, A., Harju, V., ... & Fox, A. (2018). First report of Pepper veinal mottle virus, Pepper yellows virus and a novel enamovirus in chilli pepper (Capsicum sp.) in Rwanda. *New Disease Reports*, *37*(5), 2044-0588. |
|  | PVY |  | 2021 | Waweru, B. W., Miano, D. W., Kilalo, D. C., Rukundo, P., & Kimenju, J. W. (2021). Detection and distribution of viruses infecting hot pepper (Capsicum spp.) in Rwanda. *Journal of Plant Pathology*, *103*, 573-585. |
|  | CMV |  | 2018 | Skelton, A., Uzayisenga, B., Fowkes, A., Adams, I., Buxton-Kirk, A., Harju, V., ... & Fox, A. (2018). First report of Pepper veinal mottle virus, Pepper yellows virus and a novel enamovirus in chilli pepper (Capsicum sp.) in Rwanda. *New Disease Reports*, *37*(5), 2044-0588. |
|  | PeVYV |  | 2018 | Skelton, A., Uzayisenga, B., Fowkes, A., Adams, I., Buxton-Kirk, A., Harju, V., ... & Fox, A. (2018). First report of Pepper veinal mottle virus, Pepper yellows virus and a novel enamovirus in chilli pepper (Capsicum sp.) in Rwanda. *New Disease Reports*, *37*(5), 2044-0588. |
| Ethiopia | PVMV |  | 1993 | Agranovsky, A. A. (1993). Virus diseases of pepper (Capsicum annuum L.) in Ethiopia. *Journal of phytopathology*, *138*(2), 89-97. |
|  | ChiVMV |  | 2001 | Dafalla, G. A. (2001). Situation of tomato and pepper viruses in Africa. |
|  | PVY |  | 1993 | Agranovsky, A. A. (1993). Virus diseases of pepper (Capsicum annuum L.) in Ethiopia. *Journal of phytopathology*, *138*(2), 89-97. |
|  | CMV |  | 1993 | Agranovsky, A. A. (1993). Virus diseases of pepper (Capsicum annuum L.) in Ethiopia. *Journal of phytopathology*, *138*(2), 89-97. |
| Uganda | PVMV |  | 2001 | Dafalla, G. A. (2001). Situation of tomato and pepper viruses in Africa |
|  | ChiVMV |  | 2001 | Dafalla, G. A. (2001). Situation of tomato and pepper viruses in Africa |
|  | PVY |  | 2001 | Dafalla, G. A. (2001). Situation of tomato and pepper viruses in Africa |
|  | CMV |  | 2001 | Dafalla, G. A. (2001). Situation of tomato and pepper viruses in Africa |
| Kenya | PVMV |  | 1993 | Agranovsky, A. A. (1993). Virus diseases of pepper (Capsicum annuum L.) in Ethiopia. *Journal of phytopathology*, *138*(2), 89-97. |
|  | PVY |  | 2001 | Dafalla, G. A. (2001). Situation of tomato and pepper viruses in Africa |
|  | CMV |  | 1975 | Bock, K.R., Guthrie, E.J. and Pearson, M.N. 1975. Notes on East African plant virus diseases 9: Cucumber mosaic virus. East African Agricultural and Forestry Journal, 41: 81-84 |
| Tanzania | PVMV |  | 2001 | Dafalla, G. A. (2001). Situation of tomato and pepper viruses in Africa |
|  | ChiVMV |  | 1991 | Dafalla, G. A. (2001). Situation of tomato and pepper viruses in Africa |
|  | PVY |  | 1991 | Dafalla, G. A. (2001). Situation of tomato and pepper viruses in Africa |
|  | CMV |  | 2001 | Dafalla, G. A. (2001). Situation of tomato and pepper viruses in Africa |
| South Africa | PVMV |  | 1978 | Brunt, A. A., Kenten, R. H., & Phillips, S. (1978). Symptomatologically distinct strains of pepper veinal mottle virus from four West African solanaceous crops. *Annals of Applied Biology*, *88*(1), 115-119. |
|  | PVY |  | 1996 | Budnik, K., Laing, M. D., & Da Graca, J. V. (1996). Reduction of yield losses in pepper crops caused by Potato Virus Y in KwaZulu-Natal, South Africa, using plastic mulch and yellow sticky traps. *Phytoparasitica*, *24*, 119-124. |
|  | CMV |  | 1996 | Budnik, K., Laing, M. D., & Da Graca, J. V. (1996). Reduction of yield losses in pepper crops caused by Potato Virus Y in KwaZulu-Natal, South Africa, using plastic mulch and yellow sticky traps. *Phytoparasitica*, *24*, 119-124. |
| Tunisia | PVMV |  | 1999 | Gorsane, F., Fakhfakh, H., Tourneur, C., Makni, M., & Marrakchi, M. (1999). Some biological and molecular properties of pepper veinal mottle virus isolates occurring in Tunisia. *Plant Molecular Biology Reporter*, *17*, 149-158. |
|  | PVY |  | 1994 | Gorsane, F., Fakhfakh, H., Tourneur, C., Makni, M., & Marrakchi, M. (1999). Some biological and molecular properties of pepper veinal mottle virus isolates occurring in Tunisia. *Plant Molecular Biology Reporter*, *17*, 149-158. |
|  | CMV |  |  |  |
|  | PeVYV |  | 2013 | Buzkan, N., Arpaci, B. B., Simon, V., Fakhfakh, H., & Moury, B. (2013). High prevalence of poleroviruses in field-grown pepper in Turkey and Tunisia. *Archives of virology*, *158*, 881-885. |
| Egypt | PVY |  | 2015 | El Banna, O. H., Hassan, A., & Hamed, A. H. (2015). Biological, cytopathological and molecular studies of Potato virus Y isolated from pepper grown under greenhouse conditions in Egypt. *IJ Sci. & Eng. Res*, *6*(10), 1281-1289. |
|  | CMV |  | 1987 | Khalil, E. M., & Mikhail, M. S. (1987). The use of enzyme-linked immunosorbent assay (ELISA) as a rapid and quantitative detection method for cucumber mosaic virus (CMV) in peppers. |
| Malawi | PVY |  | 2001 | Dafalla, G. A. (2001). Situation of tomato and pepper viruses in Africa |
|  | CMV |  | 2001 | Dafalla, G. A. (2001). Situation of tomato and pepper viruses in Africa |
| Zimbabwe | PVY |  | 2016 | Karavina, C., Ximba, S., Ibaba, J. D., & Gubba, A. (2016). First report of a mixed infection of Potato virus Y and Tomato spotted wilt virus on pepper (Capsicum annuum) in Zimbabwe. *Plant disease*, *100*(7), 1513-1513. |
| Zambia | PVY |  | 1999 | Ndunguru, J., & Kapooria, R. G. (1999). Identification and incidence of virus diseases of Capsicum annuum in the Lusaka Province of Zambia. *EPPO Bulletin*, *29*(1‐2), 183-189. |
|  | CMV |  | 1999 | Ndunguru, J., & Kapooria, R. G. (1999). Identification and incidence of virus diseases of Capsicum annuum in the Lusaka Province of Zambia. *EPPO Bulletin*, *29*(1‐2), 183-189. |
| Madagascar | PVY |  |  | Dafalla, G. A. (2001). Situation of tomato and pepper viruses in Africa |
|  | CMV |  |  | Dafalla, G. A. (2001). Situation of tomato and pepper viruses in Africa |
| Morocco | CMV |  | 1976 | Lockhart, B. E. L., & Fischer, H. U. (1976). CUCUMBER MOSAIC VIRUS INFECTIONS OF PEPPER IN MOROCCO. *The Plant Disease Reporter*, *60*(1-6), 262. |
| Sudan | PeVYV |  | 2014 | Alfaro-Fernández, A., ElShafie E., Ali M., El Bashir O., Córdoba-Sellés M.,Ambrosio M. F. S. (2014). First report of Pepper vein yellows virus infecting hot pepper in Sudan. Plant Disease 98, 1446-1446. doi: <https://doi.org/10.1094/PDIS-03-14-0251-PDN>. |
|  | CMV |  | 2001 | Dafalla, G. A. (2001). Situation of tomato and pepper viruses in Africa |
